# Using machine learning to identify frequent attendance at accident and emergency services in Lanarkshire

**DOI:** 10.1177/20552076251315293

**Published:** 2025-03-02

**Authors:** Fergus Reid, S. Josephine Pravinkumar, Roma Maguire, Ashleigh Main, Haruno McCartney, Lewis Winters, Feng Dong

**Affiliations:** 1 Computer and Information Sciences, 3527 University of Strathclyde, Glasgow, UK; 2 3077 NHS Lanarkshire, Bothwell, South Lanarkshire, UK

**Keywords:** Frequent attendance, accident and emergency, risk factors, machine learning, public health

## Abstract

**Background:**

Frequent attenders to accident and emergency (A&E) services pose complex challenges for healthcare providers, often driven by critical clinical needs. Machine learning (ML) offers potential for predictive approaches to managing frequent attendance, yet its application in this area is limited. Existing studies often focus on specific populations or models, raising concerns about generalisability. Identifying risk factors for frequent attendance and high resource use is crucial for effective prevention strategies.

**Objectives:**

This research aims to evaluate the strengths and weaknesses of ML approaches in predicting frequent A&E attendance in NHS Lanarkshire, Scotland, identify associated risk factors and compare findings with existing research to uncover commonalities and differences.

**Method:**

Health and social care data were collected from 17,437 A&E patients in NHS Lanarkshire (2021–2022), including clinical, social and demographic information. Five classification models were tested: multinomial logistic regression (LR), random forests (RF), support vector machine (SVM) classifier, k-nearest neighbours (k-NN) and multi-layer perceptron (MLP) classifier. Models were evaluated using a confusion matrix and metrics such as precision, recall, F1 and area under the curve. Shapley values were used to identify risk factors.

**Results:**

MLP achieved the highest F1 score (0.75), followed by k-NN, RF and SVM (0.72 each), and LR (0.70). Key health conditions and risk factors consistently predicted frequent attendance across models, with some variation highlighting dataset-specific characteristics.

**Conclusions:**

This study underscores the utility of combining ML models to enhance prediction accuracy and identify risk factors. Findings align with existing research but reveal unique insights specific to the dataset and methodology.

## Introduction and background

The Royal College of Emergency Medicine Best Practice Guidelines^1^ notes that ‘frequent attendance’ (FA) is most commonly defined as attendance of an emergency department five or more times per year. However, FA is a loosely-defined term which can vary between facilities, service type and other factors.^1^ When compared to similar population groups, these patients often require extensive medical resources and attention, Furthermore, FA is often indicative of underlying issues such as chronic illnesses, mental health problems or socio-economic challenges that are not adequately addressed through emergency services alone. It is recognised that those frequently attending accident and emergency (A&E) services also tend to use other health and social care facilities frequently.^2^ These highlight the need for effective approaches to identify and manage these high-risk patients, enabling healthcare providers to implement targeted interventions that can reduce A&E visits and improve overall patient outcomes.

Machine learning (ML) models have a great potential to identify health risk factors. MLs are a class of predictive algorithms that leverage mathematics and statistics to learn patterns from data to make predictions or decisions. By training on historical data, ML models can generalise from past examples to predict future outcomes, identify trends and uncover insights that might not be immediately obvious to humans. By analysing vast amounts of patient data, such as of electronic health records (EHRs), ML algorithms can achieve unprecedented accuracy compared to traditional methods in a wide range of clinical applications, for example, heart attacks and strokes^3^ and breast cancer.^4^ These predictions help in early diagnosis, targeted interventions, personalised treatment plans and proactive health management.^5^

Using ML models to identify risks associated with frequent and high resource use of A&E services fulfils the demand for a more anticipatory and predictive approach to the identification and management of FA – moving from a system that is reactive, dealing with events once thresholds are met, to one that can identify a person's risk of FA or even high resource use early in their trajectory. Such intelligence provides useful information to facilitate better preventive care and resource allocation, leading to early management to reduce unscheduled service use for these individuals.

However, the use of ML in this area is largely unexplored as of yet. Despite the promising results demonstrated by some of the early work, several limitations must be acknowledged: (a) firstly, the majority of existing studies have experimented with only a limited range of ML models. Typically, each study has focused on a single model, many of which use logistic regression.^^6,7,8,9^^ Other potentially very powerful models such as support vector machines (SVMs) and decision trees were only used to a very limited extent.^10^ In particular, the use of deep neural networks for FA risk identification is rare. These limitations restrict the ability to compare the effectiveness of different algorithms; (b) secondly, most studies are conducted on specific populations in emergency services, for example, geographic areas such as Norway,^7^ Yorkshire^8^ or Quebec,^9^ or GP practice,^7^ raising concerns about the generalisability of their findings to other populations such as people in Scotland. The demographic and clinical characteristics of the study populations in Lanarkshire or other geographic locations can vary widely from other previously studied populations, making it difficult to apply the results universally; and (c) lastly, while some common risk factors for frequent A&E attendance have been identified across the aforementioned studies, there are notable differences as well. Factors such as chronic conditions, mental health issues and socio-economic challenges are frequently cited, but the relative importance and interaction of these variables can vary. In particular, it is not clear whether the population in Lanarkshire share these common risk factors. These variations highlight the need for this research.

Correspondingly, the aim of this research has therefore been to investigate ML approaches for predicting FA of the A&E services in the National Health Service (NHS) in Lanarkshire, Scotland. Our research has sought to answer the following research questions:

- What are the strengths and limitations of different ML models for predicting individual risk of FA? Understanding the strengths and limitations of various ML models, such as logistic regression, decision trees, random forests (RF), SVMs, k-nearest neighbours (k-NN) and neural networks, through evaluation with key performance indicators such as accuracy, precision, recall, F1 score and area under the curve (AUC), provides critical evidence for healthcare providers to select the most appropriate and robust model that best suits the clinical scenarios and demands.
- What are the common risk factors among patients, and is there a consensus between different models? This inquiry helps uncover the underlying causes of frequent A&E attendance and high resource use. By analysing risk factors such as age, alcohol consumption, substance use, chronic conditions, socio-economic status, mental health issues and previous medical history, we can gain insights into the drivers of high A&E usage. Moreover, comparing the risk factors identified by different ML models allows us to verify the consistency and reliability of the predictions. A consensus on risk factors across models enhances the credibility of the findings.
- Are the results of this research comparable with existing research? This research question seeks to determine whether the predictive models and identified risk factors align with results found in prior research. Comparing the results of this research with existing studies contextualises the findings and validates the models used. Through these comparisons, we assess the generalisability and applicability of our findings to different populations and settings. Furthermore, it allows us to identify any novel insights or discrepancies that may warrant further investigation.

The main contributions are: (a) this study is specifically tailored to the Lanarkshire population in Scotland, with a focus on identifying those attending A&E departments frequently. By concentrating on this distinct demographic, we aim to provide insights that are directly relevant, applicable and beneficial to the local healthcare providers to address the unique challenges faced by the A&E services, particularly in managing and allocating resources more effectively; (b) unlike many previous studies that typically employ a single ML model, our research encompasses a spectrum of ML models, including logistic regression, RF, SVM, k-NN and neural networks. By evaluating multiple models, we compare their performance rigorously and identify the most effective algorithms. Our particular focus is on exploring the potential of neural network models. Given that deep learning is currently a dominant force in the field of ML,^^11^^ we investigate its capabilities in handling complex, high-dimensional data, especially in the scenario where the data are imbalanced; (c) we provide a computationally efficient method for Shapley value^^12^^ approximation to rank the feature importance. Shapley values offer a robust framework for interpreting complex ML models by distributing the predictive power of each feature in a fair and consistent manner. This is applied across the five models, allowing us to compare and reach a consensus on the most significant risk factors. This approach helps us identify the key risk factors that lead to frequent A&E attendance and high resource use. Pinpointing these critical factors allows the possibility to design targeted prevention methods to mitigate the risks; and (d) we compared our ML model performance and risk factor identification outcomes with existing research focused on Scotland to identify commonalities and differences. By doing so, we aimed to validate our findings and understand how our study aligns with or diverges from prior work in the region. This comparison helps to contextualise our results within the broader landscape of healthcare research in Scotland, providing valuable insights into the consistency and uniqueness of our findings. Overall, this research provides further understanding of the effectiveness of ML models in predicting frequent A&E attendance and high resource use, the common risk factors involved and the alignment of our findings with existing research. This approach ensures a thorough examination of the issue, facilitating the development of targeted interventions to reduce frequent A&E visits and improve patient care.

## Related work

This section provides an overview of the related work in identifying risk factors for FA in health services. We present the important variables involved in the previous studies and also review the work conducted in Scotland.

### ML for risk identification of FA

Studies involving the use of ML were few in the area of risk detection for FA. The most popular method thus far is logistic regression. Examples with logistic regression include those outlined in section ‘Introduction and background’.^^6,7,8,9^^ Other studies involving decision trees also exist, but the results from these studies varied, with the ML models giving equal or worse performance than logistic regression when tasked with classification of future frequent attendance.^^10,13^^

In one study,^14^ a tree-based method was used, namely XGBoost. The model was trained using five-fold cross-validation, and the test set was defined using 95% confidence intervals, which were calculated using bootstrapping. Data in this study were collected from Southampton's Emergency Department (University Hospitals Southampton Foundation Trust) from 1 April 2019 to 30 April 2020. During training, hyperparameters were selected using a Tree Parzen Estimator. The most favourable results in this study were from XGBoost, which achieved an AUCROC of 0.747. However, during testing, the model only achieved a precision of 0.233. This suggests that, while the model's overall predictive capacity is moderate to high, it is likely overfitted. This study differs from the study undertaken here in that it examines only the likelihood of a patient reattending an emergency department (ED) within 30 days of an initial visit, thus making a direct comparison challenging. However, it can help to provide some context of model selection and results.

A second study^13^ which investigated the prediction of attendances to A&E departments also utilised gradient-boosted trees, namely AdaBoost, as well as standard decision trees. This study also used logistic regression as a baseline model against which the results of the two tree-based models were compared. The data were collected from a non-public dataset from the California Office of State-wide Health Planning and Development (OSHPD) covering the years 2009–2013, and from this dataset, four cohorts of patients with one or more ED visits were constructed for the years 2009, 2010, 2011 and 2012, respectively. Models were trained on the 2009 cohort and tested on cohorts from subsequent years. The authors sought to use ML methods for two classification tasks: a three-class classification task, where patients were classified as either low-frequency (<1 visit), medium-frequency (2–4 visits) and high-frequency (≥5 visits) ED users, and a second binary classification task, where patients were classified as either low-frequency (<*p* visits) or high-frequency (≥*p* visits) ED users. The threshold for p was varied between 2 and 9, and models were trained for each threshold. Results varied from cohort to cohort. For the three-class classification task, the study only reports detailed metrics for performance on specific classes, rather than aggregated performance across all classes, meaning that only a basic average of results across all classes can be calculated post hoc. For the binary classification task, results are only presented in a line plot, making exact analysis of results challenging. However, from the study's results, the same problem presents itself, namely that in all but the most frequent class, precision is very low, and the models are likely overfitted to the most frequent class, very likely limiting their feasibility in real-world use. It is also worth noting that in this study, the most frequent class was in fact those with one or fewer visits, meaning that the models in this study are seemingly overfitted to predicting infrequent attendance. This would likely prevent the models from being useful in the prediction of FA.

A third study^10^ assessed the performance of nine models. The study only examined the FA of EDs of those with epilepsy, limiting the generalisability of findings. The data were collected from a health information exchange in New York City. The study used data from a 2-year period, and utilised data from year 1 to predict frequent ED attendance (≥4 visits) in year 2, thus treating the task as a binary classification. Of all methods, lasso, RF and AdaBoost all achieved high AUCROC scores. However, all of the models had poor sensitivity (<50%). This again suggests that the models are not well-generalised and have overfit to the positive class. The study ultimately proposes that a simple strategy of selecting those with >11 ED visits in year 1 as being most at risk of FA in the subsequent year outperforms most models’ predictions of those who are most at risk of continued frequent attendance. However, this strategy requires that an individual very frequently attends ED in one year to be classified as likely to frequently attend in the next year. Arguably this methodology is only useful as a strategy to predict continued high levels of FA, rather than the emergence of high levels of FA.

A final study^15^ examined the use of recurrent neural networks to predict FA based on time-series data. In the context of this study, the examination of the use of recurrent neural networks is only tangentially useful as an example of the use of deep neural networks in the prediction of FA, as our study does not examine time-series data.

### Important variables

A rapid review was conducted to find studies using any methods to investigate FA of any healthcare setting. A total of 31 studies were included (Table C.1). Most included studies reported risk factors for FA of EDs (N = 29). Two studies investigated FA of GP practices. There was significant variability in the definition of FA; definitions ranged from three to 20+ times a year (in attendance), with some studies even determining FA as the top 10% (in attendance) of a particular cohort of participants.

The variables reported by the included studies (N = 31) to be the greatest risk factors for FA were: age (N = 21), sex (N = 20), mental illness (N = 13), outcome after care (N = 8), intensity of FA (N = 7), substance abuse (N = 6), triage level (N = 6), origin of stay in hospital (N = 5), chronic illness/long-term disease (N = 5), length of stay (N = 5) and residence (i.e. low-come vs high-income area) (N = 5). Triage level, origin of stay in hospital and length of stay were not represented in this project's dataset. Additionally, only mortality was represented as an outcome after care.

Results of the included studies were in some aspects extremely heterogeneous, e.g. some studies reported females to exhibit greater frequent attendance while other studies reported the opposite or no significant differences. However, there was a general consensus between included studies that increased age was a significant risk factor for FA to healthcare services.

### Study in Scotland

A rapid review of Scottish studies investigating FA in Scotland was conducted, and very few studies were found. Of the included studies, each focused on a specific factor, such as: COVID-19, social connectedness, cancer and patient experience. Kyle et al.^16^ explored changes in FA behaviour in relation to the COVID-19 pandemic. The results concluded that both before and after the pandemic FA groups were predominantly male, and homelessness, mental health problems and substance use were commonly reported among both groups. It also highlighted that FA was reduced during the pandemic. There were some inconsistencies between studies however, as Kyle et al.^^16^^ reported that most FA patients lived in socially deprived areas, which is contrary to the findings of a previous study,^17^ who found no association and reported that socio-economic circumstance is not associated with FA when the greater burden of ill health in deprived areas was considered. Cruwys et al.^18^ investigated how social connectedness impacts FA. The study reported that there is a significant association between FA and social interaction, suggesting that social isolation could be considered as a risk factor for FA in primary care. Other studies based in Scotland focused on cancer-related frequent attendance. Mills et al. (2022)^19^ reported that patients diagnosed with cancer who attended A&E frequently were more likely to be elderly, ‘have upper gastrointestinal, haematological, breast and/or ovarian malignancies’ than those attending infrequently.

Initial analysis of the 73 patients with the highest number of attendances in Lanarkshire over both 2014/15 and 2015/16 identified that overall, the majority were male (57.5%) with a higher proportion being male in South Lanarkshire (62%) compared to North Lanarkshire (53%). These data indicate that the FAs seen within Lanarkshire are predominantly a younger cohort (i.e. not frail elderly), vulnerable and facing the multiple challenges of deprivation and poor health, both physical and mental.

An analysis of FA in NHS Lanarkshire in the years 2017/18 found notable characteristics including:

- A total of 83% and 92% were under 65 years in North and South Lanarkshire, respectively.
- A total of 90% had at least one long-term condition (LTC) with 37% having more than five LTC.
- A total of 77% of those who exhibit FA lived in the most deprived areas (Scottish Index of Multiple Deprivation (SIMD) 1 and 2).
- Mental health related issues accounted for the top three reasons for ED attendance, including ‘mental health – alcohol’ and ‘mental health – feared complaint no diagnosis’. These are similar issues identified in a UK study which reviewed reasons for FAs in ED.^20^
- FAs peaked later on at night (4–9 p.m.), the general population's ED attendances peaked in the mid-morning/afternoon (11 a.m.–1 p.m.), however, time of admission was not included in the final dataset.

## The dataset

### Dataset curation

This research utilises primary and secondary health data and social care data gathered from existing patient records associated with A&E department visits in NHS Lanarkshire. The retrospective data were extracted from previously collected routine patient-level information, which contain variables potentially associated with high attendance at healthcare facilities. The patient-level dataset was provided by the Local Intelligence Support Team (LIST) in Public Health Scotland (PHS), with all identifiable variables removed. A PHS Data Release and Linkage Form was approved to authorise the release of this dataset to NHS Lanarkshire, this contains approval from the NHS Lanarkshire Caldicott Guardian, North and South Lanarkshire HSCPs’ Senior Responsible Officers and the PHS Data Protection Team. Caldicott Guardian approval was granted for named NHSL researchers’ use of patient identifiable data to ensure that it is pseudonymised, e.g. age is converted to age range and only a pseudonymised version was released for the research. No information that is directly identifiable was used for the research and we stored the de-identified data only for the purposes of this research project.

The dataset spans the period from 2021 to 2022 and provides a comprehensive overview of A&E visits within this timeframe. Those patients who had fewer than three visits to A&E departments in NHS Lanarkshire were excluded from the dataset.

The dataset covers known risk factors from previous studies including clinical, social and demographics information. Patient demographic information (age and gender), locality within local authority, SIMD and health and social care data. Health and social care data include numbers of clinical episodes (including alcohol, mental health, substance misuse, self-harm), home care episodes, clinical conditions, mortality, homeless applications were contained in the dataset. Table 1 offers a list of the variables and their descriptions. The dataset is comprised of 17,437 rows, each relating to an individual patient. Hence in total there are 17,437 patients who meet the inclusion criteria during the study period (2021–2022).

**Table 1.** List of variables contained in the dataset.

Variable name	Description
Number of AE attendances	Number of accident and emergency department attendances for 2021/22
Deceased flag	Deceased flag for 2021/22 (yes = deceased; no = alive)
Gender	Gender
Age group	Age at mid-point of financial year. In total there are six age groups ranging from 0–15 in group 1 to 75+ in group 6.
Local council authority	Local council authority of residence in 2021/22
SIMD Scotland level population-weighted quintile	SIMD 2020v2 Scotland level population-weighted quintile (1 = most deprived; 5 = least deprived) in 2021/22
Homeless application flag	Patient had an active homelessness application during financial year 2021/22
Number of home care episodes	Total number of home care episodes, includes personal, non-personal and unknown type in 2021/22
Number of acute inpatient episodes	Number of acute inpatient episodes in 2021/22
Number of mental health inpatient episodes	Number of mental health inpatient episodes 2021/22
Self-harm episodes	Sum of episode flags for self-harm admission or attendance in 2021/22
Substance misuse episodes	Sum of episode flags for substance misuse admission or attendance in 2021/22
Alcohol episodes	Sum of episode flags for alcohol admission or attendance in 2021/22
Arthritis LTC	Arthritis LTC marker in 2021/22 No = no diagnosis made Yes = diagnosed with this LTC
Respiratory LTC	Asthma LTC marker in 2021/22, chronic obstructive pulmonary disease (COPD) LTC marker in 2021/22 No = no diagnosis made Yes = diagnosed with 1+ of these LTCs
Cardiac LTC	Atrial fibrillation LTC marker in 2021/22, heart failure LTC marker in 2021/22, coronary heart disease (CHD) LTC marker in 2021/22 No = no diagnosis made Yes = diagnosed with 1+ of these LTCs
Cancer LTC	Cancer LTC marker in 2021/22 No = no diagnosis made Yes = diagnosed with 1+ LTCs
Cerebrovascular disease LTC	Cerebrovascular disease (CVD) LTC marker in 2021/22 No = no diagnosis made Yes = diagnosed with 1+ LTCs
Digestive LTC	Chronic liver disease LTC marker in 2021/22, other diseases of digestive system flag in 2021/22 No = no diagnosis made Yes = diagnosed with 1+ of these LTCs
Neurological LTC	Dementia LTC marker in 2021/22, epilepsy LTC marker in 2021/22, multiple sclerosis LTC marker in 2021/22, Parkinsons LTC marker in 2021/22 No = no diagnosis made Yes = diagnosed with 1+ of these LTCs
Diabetes, other endocrine and metabolic LTC	Diabetes LTC marker in 2021/22, other endocrine metabolic diseases LTC marker in 2021/22 No = no diagnosis made Yes = diagnosed with 1+ of these LTCs
Renal failure LTC	Renal failure LTC marker in 2021/22 No = no diagnosis made Yes = diagnosed with 1+ LTCs
Other LTC	Congenital problems LTC marker in 2021/22, diseases of blood and blood forming organs LTC marker in 2021/22 No = no diagnosis made Yes = diagnosed with 1+ of these LTCs
Total number of LTCs	Sum of individual LTCs for patient in that row 2021/22 (arthritis LTC marker, asthma LTC marker, chronic obstructive pulmonary disease (COPD) LTC marker, atrial fibrillation LTC marker, heart failure LTC marker, coronary heart disease (CHD) LTC marker, cancer LTC marker, cerebrovascular disease (CVD) LTC marker, chronic liver disease LTC marker, other diseases of digestive system flag, dementia LTC marker, epilepsy LTC marker, multiple sclerosis LTC marker, Parkinsons LTC marker, diabetes LTC marker, other endocrine metabolic diseases LTC marker, renal failure LTC marker, congenital problems LTC marker, diseases of blood and blood forming organs LTC marker)
Scottish patients at risk of readmission or admission (SPARRA) 12-month risk score	SPARRA 12-month risk score from the start of the financial year of 2021/22

### Scottish patients at risk of readmission or admission (SPARRA) scores

Scottish patients at risk of readmission or admission (SPARRA) scores^21^ quantify a patient's risk of emergency hospital inpatient admission in the next 12 months. SPARRA draws from a range of variables. Hospital inpatient admissions, prescribing data, psychiatric admissions, ED admissions, outpatient attendances and demographic information are all utilised with a logistic regression model which estimates the patient's risk of emergency inpatient admission.

### SIMD

The SIMD is a publicly-available metric for small geographic areas within Scotland. The SIMD ranks small areas known as datazones from most deprived to least deprived.^22^ For the purposes of this study, SIMD population-weighted quintiles were used. Datazones are grouped into five categories known as quintiles. The population-weighted quintiles ensures a very similar population size for each quintile, with the most deprived quintile ranked 1, and the least deprived quintile ranked 5.

### Initial exploratory data analysis

The dataset reveals that the mean number of A&E visits per user is 4.14, with a standard deviation of 2.81, indicating considerable variability in the number of visits among users. Notably, the dataset includes 348 patients who attended A&E more than 10 times in 2021/22, highlighting the presence of a small subset of individuals who utilise A&E services extensively.

Initial analysis also indicates that the distribution of A&E attendances underscores the presence of a small number of high-frequency patients who account for a disproportionate number of total visits 1. The vast majority of patients have a relatively low number of visits, with the data showing a significant concentration around the lower end of the scale. Specifically, most patients in the dataset have had three or four A&E visits, with 13,474 users falling into this category. This number surpasses the combined total of patients who have between five and ten visits, which stands at 3615. Further analysis also reveals that 75% of patients have four or fewer visits to A&E. This percentile further emphasises the skewness of the dataset, where a large majority of patients have relatively few visits, while a small minority have a significantly higher number of visits.

The skewed distribution poses challenges for predictive modelling, since it predisposes the models to predicting that an individual will have fewer rather than more visits. Hence, the models need to be sensitive to the high skewness and capable of accurately identifying the small group of users who are high-frequency attenders. Addressing this challenge is crucial to predicting FA in this research. We have employed weighted data sampling to address the data imbalance – more details are presented in section ‘Methods’.

### Ethical approval

This study was approved by NHS Research Ethics Committee (Health & Care Research Wales REC 4),^31^ and the reference number for the ethical approval is (24/WA/0041).

## Methods

This study is retrospective in nature and was conducted within the NHS Lanarkshire Health Board, Scotland. It is an observational study focused on data collected over a one-year period, from year 2021 to 2022. This timeframe provides a comprehensive overview of patient attendance patterns at A&E departments within the region. The dataset used for this research was provided by Public Health Scotland's Local Intelligence Support Team (LIST) and is derived from the linked ‘SOURCE’ dataset, which includes pseudonymised CHI numbers. NHS staff removed all personally identifiable information before sharing the data with the data scientists, ensuring compliance with appropriate information governance and data protection standards, as the study involves the secondary use of previously collected routine data without requiring patient consent. The SOURCE dataset encompasses health and social care information, including factors potentially associated with FA at healthcare facilities – see section ‘The dataset’ for more details. A pseudonymised dataset was securely transferred for analysis. The inclusion and exclusion criteria are described below.

**Figure 1.**
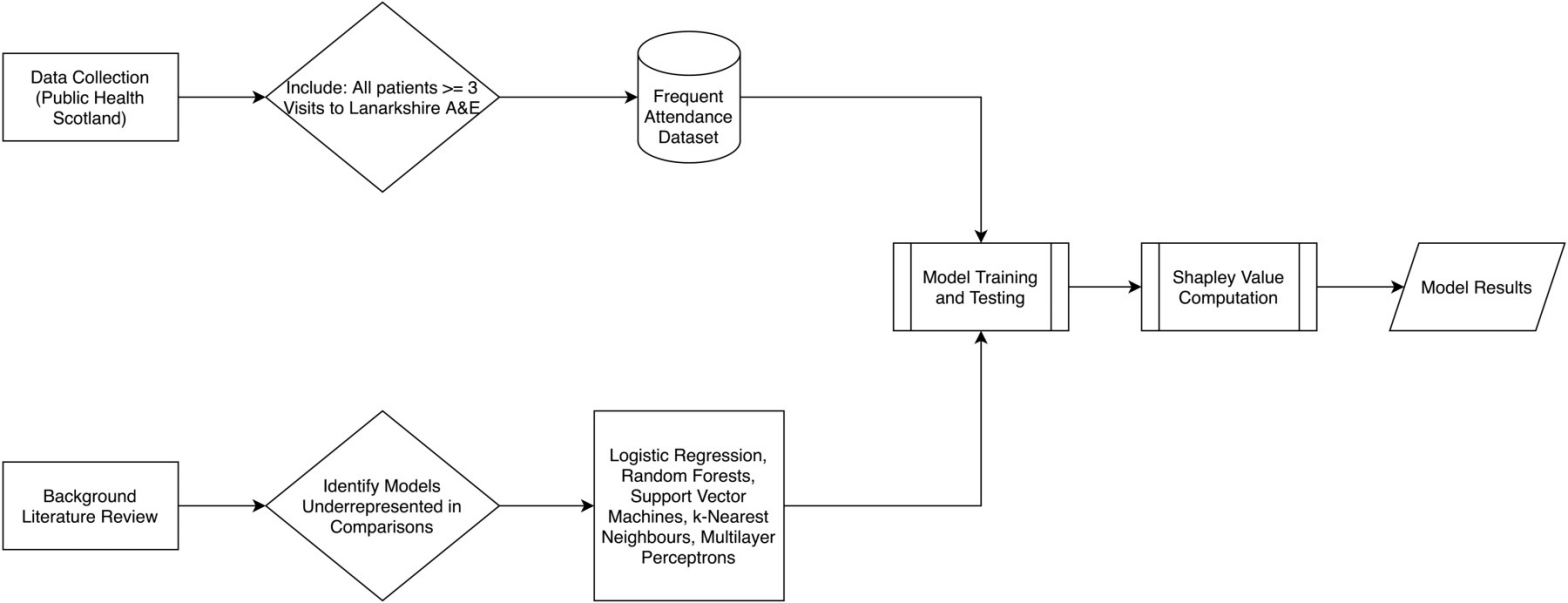
Block diagram detailing the research process.

The above image (Figure 1) demonstrates the research process of this study. Alongside our background literature review, data were collected by Public Health Scotland from their SOURCE dataset, as detailed in the previous paragraph. The dataset was filtered to include patients who had been admitted to NHS Lanarkshire A&E departments three or more times and exclude those who had been admitted fewer than three times. The result was our frequent attendance dataset – see section ‘The dataset’ for more details. Our background literature review identified the models assessed in this paper as being underrepresented in existing research on FA. We then utilise the frequent attendance dataset to train and test the models identified in this stage of the research. Using these trained models, we can then compute Shapley value approximations for each model. The final results of the training and testing, as well as the Shapley values, can then be used to compare model performance and identification of risk factors.

### Inclusion and exclusion criteria

This study is retrospective in nature. Data were collected from all patients in NHS Lanarkshire health board who had attended A&E on at least three occasions during 2021/22. All patients who met this criterion were included. Patients with fewer attendances were excluded as they are less likely to exhibit patterns indicative of recurrent or chronic conditions requiring frequent A&E visits. By focusing on this cohort, this study seeks to understand the trajectory of individuals who have reached this threshold and assess how they might progress to even higher attendance levels. This knowledge is critical for developing preventative strategies aimed at reducing A&E visits, with a particular focus on intervening at the three-visit threshold to potentially stabilise or reduce the frequency of future attendance. We are particularly interested in identifying significant risk factors associated with FA, providing insights into how these factors contribute to high A&E use.

### Approach selection

The distribution of A&E visits in the dataset presents a challenge for regression analysis due to the high variability and skewness in the number of visits among patients. One primary issue with regression in this context is the difficulty in finding a model that can effectively represent such a large and diverse group of patients who share the same number of AE visits. For instance, there are 9730 users with exactly three A&E visits, and similarly, a considerable number of patients fall into categories with four or five visits. This homogeneity in visit counts among a substantial number of patients introduces noise and reduces the model's predictive power. A regression model would struggle to account for the underlying differences in patient characteristics that lead to the same visit count, thus leading to less accurate and meaningful predictions. Moreover, the presence of outliers exacerbates the challenge for regression models. These extreme values can disproportionately influence the model, causing it to perform poorly for the majority of patients who have a much lower number of visits. The skewed distribution and the wide range of visit counts further diminish the regression model's ability to generalise across different patient groups.

On the other hand, classification models offer a more effective alternative. By categorising patients into discrete groups based on their A&E visit frequency, classification models can handle the variability and skewness more robustly. Instead of predicting the exact number of visits, a classification model can predict whether a patient is a low-frequency attender, medium-frequency attender or high-frequency attender. This approach simplifies the modelling process and enhances the model's ability to provide actionable insights. Using classification, we can better manage and interpret the data, as the model focuses on distinguishing between categories rather than fitting a precise number for each patient. This method is particularly advantageous when dealing with large groups of patients who share the same number of visits, as it allows us to leverage the commonalities within each category while accounting for individual variations.

### Data cleaning and pre-processing

#### Variable removal

Some data cleaning was undertaken to remove interdependence between variables. Two variables were removed from the dataset: SPARRA 12-month risk score and local authority. This decision was made due to the fact that SPARRA scores are calculated using many of the variables which are already present in the dataset, while SIMD and local authority both relate to information about geographic areas. The decision was made to keep SIMD over local authority as it was thought that SIMD would provide more granular information.

It was found that SPARRA 12-month risk score had 1398 null values. As this variable was removed entirely, no action was required to address this.

#### Binning for classification models

Classification models aim to predict which class a record belongs to. As such, it is necessary to split the target variable into classes. The lower bound of the definition of FA is three visits in a 12-month period. However, the most commonly accepted definition of FA is five or more visits in a 12-month period.^1^ Meanwhile, High Intensity Use is defined by NHS East London as 10 or more visits to A&E in a 12-month period.^23^ These boundaries were used to inform the classification of patients into one of the three categories:

- Low attendance: number of A&E attendances < 5: Class 0, n = 13,474
- Mid attendance: number of A&E attendances ≥ 5, ≤10: Class 1, n = 3615
- High attendance: number of A&E attendances > 10: Class 2, n = 348

This allows the model to predict whether a patient is likely to belong to Class 0, 1 or 2.

### Classification models

#### Challenges

While classification models are better suited for this dataset, they need to overcome the problem arising from the high degree of feature entanglement in the dataset. It requires sophisticated modelling techniques that can navigate the complex relationships and still provide reliable and interpretable results.

To illustrate this, a t-SNE (t-distributed stochastic neighbour embedding) model^24^ was used to investigate the entanglement of features within our dataset. t-SNE is a popular dimensionality reduction technique primarily used for visualising high-dimensional data. It is particularly effective in preserving the local structure of the data, making it an excellent tool for visualising clusters or groups in complex datasets. The t-SNE results reveal that the features are highly entangled, with no clear boundaries between groups of patients. Figure 1 shows colour coded data points according to their class (i.e. 0, 1 or 2) from our dataset.

The high degree of feature entanglement indicates that the underlying relationships between patient characteristics and their A&E visit frequencies are intricate and interwoven. This complexity can stem from various factors that do not neatly categorise into distinct groups. Highly entangled features mean that models are less able to statistically separate records by drawing boundaries between groups of similar and dissimilar examples. The entanglement adds a significant layer of complexity to the task. The entanglement also complicates the interpretation of model outputs. When features are highly entangled, it is harder to pinpoint which specific factors contribute most to the prediction, making it challenging to identify the risk factors from the model.

### Models

In this study, five main classification models were used:

- Multinomial logistic regression;
- Decision trees and RFs;
- Support vector machine classifier;
- k-Nearest neighbours classifier; and
- Multi-layer perceptron (MLP) classifier.

**Figure 2.**
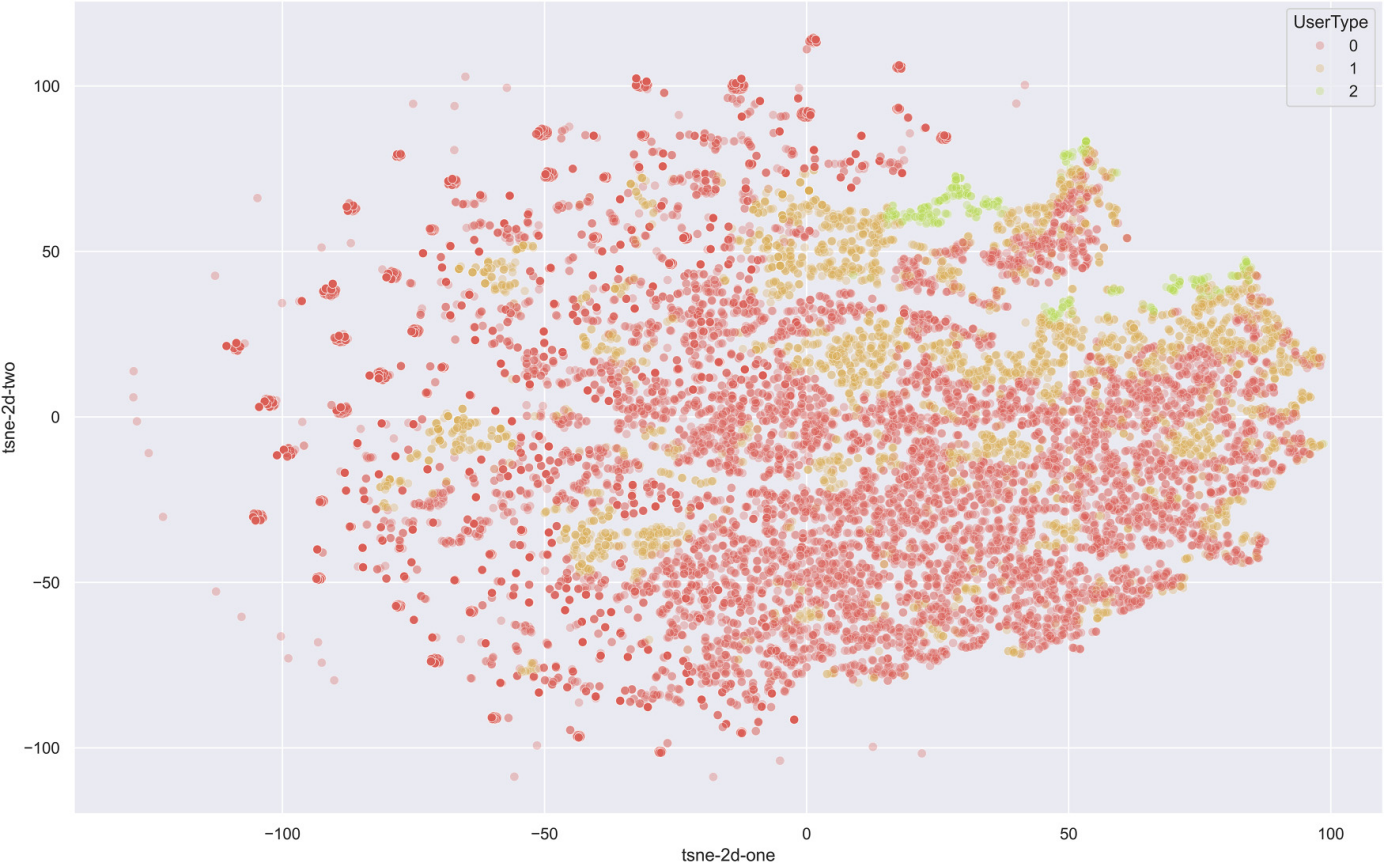
Two-dimensional t-SNE plot.

The above models were chosen based on both their usage in existing literature in this research area as outlined in section ‘Related work’, as well as their widespread usage in classification tasks in general. The selection of these models was guided by their prevalence in existing literature within this research area, as outlined in section ‘Related work’, as well as their general applicability and success in classification tasks. The combination of multinomial logistic regression, decision trees, RF, SVM, k-NN and MLP classifier provides a comprehensive set of tools to address the classification problem at hand. These models represent a spectrum from simple, interpretable models to complex, powerful models, allowing us to balance accuracy, interpretability and computational efficiency. This diverse selection ensures robust evaluation and provides a strong foundation for understanding the risk factors associated with frequent A&E attendance (Figure 2).

### Data sample weighting

To handle data imbalance, we count the number of data samples in each class and weight the importance of each sample inversely proportional to the class frequency. This means that classes with fewer samples are assigned higher weights, while those with more samples receive lower weights. This approach ensures that the model pays more attention to the minority classes during training, helping to mitigate the bias toward the majority class and improving overall performance on imbalanced datasets.

#### Multinomial logistic regression

Logistic regression (LR) in its original form is designed for binary classification tasks.^25^ Multinomial logistic regression, also known as softmax regression, directly handles multi-class classification by extending the logistic function to multiple classes. It calculates the probability that an observation belongs to each class based on a set of independent variables, and then assigns the class with the highest probability.

By including multinomial logistic regression, we ensure that our analysis begins with a method that offers high interpretability and simplicity, serving as a clear benchmark for more complex models. Its ability to provide direct insights into the influence of individual features on the likelihood of frequent A&E attendance is invaluable for healthcare providers who need to make data-driven decisions.

One of the main advantages of logistic regression is its interpretability. The coefficients in the model can be directly interpreted as the change in the log odds of the outcome for a one-unit increase in the predictor variable, holding other variables constant. This makes it easy to understand the influence of each feature on the probability of the outcome.

While logistic regression is powerful and interpretable and has been widely used in the studies of identifying risks of FA,^^6,7,8,9^^ it has limitations. It assumes a linear relationship between the log odds of the outcome and the predictor variables, which may not hold in all cases. Additionally, logistic regression can struggle with multicollinearity among predictors and is less effective with very large and complex datasets compared to more advanced techniques like decision trees or neural networks.

In this work, the logistic regression implementation^26^ used Limited Memory Broyden–Fletcher–Goldfarb–Shanno (LMBFGS) in the optimisation for training of the multinomial logistic regression model, with the maximum iterations set to 100,000 to ensure convergence. LMBFGS is a special implementation of the Broyden–Fletcher–Goldfarb–Shanno optimisation method^27^ which aims to reduce memory usage. This algorithm uses the gradient of the logistic regression cost function to approximate the inverse Hessian matrix, thus determining the search direction. The Limited Memory variant of this algorithm only stores a limited number of pairs of parameter updates and gradient differences, rather than a full history. This preserves memory and is more practical from a computational point of view when considering larger problems.

#### Decision trees and RF

A decision tree^28^ is a popular ML algorithm used for both classification and regression tasks. It works by recursively splitting the data into subsets based on the values of input features. Each internal node of the tree represents a decision point based on a specific feature, and each leaf node represents a predicted outcome. The paths from the root to the leaf nodes represent classification rules. RF^28^ is an ensemble learning method that builds upon the decision tree algorithm. It combines the predictions of multiple decision trees to produce a more robust and accurate model. The idea is to create a ‘forest’ of decision trees, each trained on a different random subset of the data and using a random subset of features at each split.

Both decision trees and RF are powerful ML models. Decision trees offer simplicity and interpretability, making them suitable for exploratory analysis and applications where model transparency is crucial. However, decision trees are prone to overfitting, especially when the tree is too deep and captures noise in the training data. RF, with their ensemble approach, provide enhanced predictive performance and robustness, making them ideal for more complex and demanding tasks. Understanding the strengths and limitations of each method is key to selecting the appropriate algorithm for a given problem. While RF are powerful and versatile, they have several limitations that practitioners need to be aware of. These include challenges with interpretability, potential for overfitting, difficulties with high-dimensional data, sensitivity to noisy data, biases in feature importance, slow prediction times and struggles with imbalanced datasets. Understanding these limitations is crucial for effectively applying RF and interpreting their results.

The decision trees and RF add a layer of complexity and robustness to our model suite. Decision trees’ ease of interpretation complements logistic regression, while their ability to capture non-linear relationships and interactions between features enhances our analytical depth. RF, as an ensemble method, mitigate the risk of overfitting inherent in individual decision trees, thus providing more reliable and generalisable results.

In this work, the random forests implementation^26^ involves the main tuneable hyperparameters for this class are the number of estimators (n_estimators), and the split criterion (criterion). The number of estimators sets the number of trees in the forest, and this was set at the default of 100. Increasing the number of trees did not produce significant changes in results. The two possible selections for split criterion are Gini Impurity (‘gini’), and Information Entropy (‘entropy’). Using Information Entropy did not produce significant changes in weighted F1 score, and so the default setting of Gini Impurity was used, as this method is less computationally expensive.

#### SVM

SVM^28^ is a powerful supervised ML algorithm used for both classification and regression tasks. It is particularly well-suited for classification problems and is known for its effectiveness in high-dimensional spaces. The core idea behind SVM is to find a hyperplane that maximises the margin between the classes. The margin is defined as the distance between the hyperplane and the nearest points from each class, which are known as support vectors. By maximising this margin, SVM ensures that the model has the best possible generalisation ability to classify unseen data points. In the case of linearly separable data, the SVM algorithm finds the hyperplane that separates the classes with the maximum margin. However, for non-linearly separable data, SVM can still be effective by employing a technique known as the kernel trick. Features which are not linearly separable can still be separated by a hyperplane if the features are first transformed to a higher dimensional space. However, this is often computationally expensive, especially when the dimensionality of the transformed space is very high or infinite, such as with the use of the radial basis function (RBF). The kernel trick method seeks mainly to solve the high computational cost of computing a complete mapping of inner products between features in a high-dimensional space. Rather than explicitly calculating a transformation of the data to a higher dimensional space and then taking the inner product between *x* and *y* in that higher dimensional space, a kernel function which is equivalent to the inner products between *x* and *y* after transformation can be computed without explicitly transforming the features. This significantly reduces the computational cost.

The inclusion of the support vector machine classifier addresses the need for a robust method capable of handling high-dimensional feature spaces and non-linear separations. SVM's use of kernel functions enables us to transform and manage complex data structures effectively. This model's strength lies in its capacity to generalise well to unseen data, which is critical in the context of predicting healthcare outcomes.

The kernel chosen for the SVM-C model was the RBF. This kernel was chosen based on the two-dimensional t-SNE plot, which showed that features were highly entangled with no clear linear separations. The RBF kernel separates features by implicitly computing their relationship in infinite-dimensional space, thus potentially finding non-linear relationships which are not accessible to other kernel methods. A five-fold grid search was performed to set the C and gamma hyperparameters of the model, using F1 score as a criterion. However, upon using the best parameters from the grid search (C = 10, gamma = 0.01), AUCROC performance was degraded significantly (default = 0.69, best parameters = 0.50). As a result, the default settings were instead used.

#### MLP

A MLP classifier^29^ is a type of artificial neural network used for supervised learning tasks, particularly classification. It is one of the simplest forms of neural networks and consists of multiple layers of nodes (neurons) connected in a feedforward manner. MLPs are known for their ability to model complex relationships in data, making them a powerful tool for various classification problems. A MLP is the most basic form of deep neural network. Deep neural networks are an extension of the MLP architecture and are characterised by a large number of hidden layers. This increased depth allows DNNs to learn hierarchical representations of data, where each successive layer captures increasingly abstract features.

The MLP classifier introduces advanced deep learning capabilities into our study. MLPs’ ability to model intricate, non-linear relationships through multiple hidden layers and activation functions positions them at the cutting edge of predictive modelling. Their flexibility and power make them particularly suitable for capturing the complex dynamics of frequent A&E attenders, which might be overlooked by simpler models. Comparing MLP performance against traditional models provides valuable insights into the added benefits and potential trade-offs of using deep learning in healthcare applications.

To address the data imbalance challenge with the MLP model, we have experimented a few approaches under the MLP setting. One of them is to experiment with a ‘value-hot’ cross entropy method. Traditional cross entropy loss, which relies on one-hot encoded ground truth labels, may not effectively handle this imbalance, often leading to suboptimal performance, especially for rare classes. The ‘value-hot cross entropy’ method modifies the conventional cross entropy loss by replacing the binary indicator (1 for the true class and 0s for others) with the actual numerical value corresponding to the ground truth class. For example, in a medical classification task predicting patient attendance numbers (e.g. A&E attendance), the ground truth label for each sample is represented by the actual attendance number rather than a binary indicator. This adjustment allows the loss function to inherently assign higher penalties to predictions that deviate from the actual numerical values, thereby prioritising the correct classification of instances with higher numerical significance. Additionally, we adjust the weighting scheme in the loss function based on the A&E visiting numbers rather than the sample frequencies. This approach ensures that the model focuses more on learning from instances that carry greater numerical importance, such as high attendance numbers, rather than solely on their occurrence frequency in the dataset.

In addition, we combine the ‘value-hot cross entropy’ method with the focal loss^30^ to enhance the robustness of classification models in handling imbalanced datasets. The focal loss, originally introduced to address the issue of foreground-background class imbalance in object detection, is adapted here to further augment the penalty for misclassifying challenging instances while integrating the numerical significance of classes through the ‘value-hot’ modification. The focal loss introduces a dynamically adjusted focusing parameter that down-weights well-classified examples and focuses more on hard-to-classify examples. This property is particularly beneficial in scenarios with severe class imbalance, where rare classes require more attention during training. By combining focal loss with ‘value-hot cross entropy’, our method enhances the model's ability to effectively learn from and prioritise challenging instances, improving overall classification performance.

To design and optimise the MLP architecture, we integrate state-of-the-art techniques such as batch normalisation to alleviate gradient issues and a rigorous ablation study to determine optimal hyperparameters, including network depth, regularisation norms (L1 or L2), dropout rates and learning rates. The MLP comprises an input layer tailored to the dimensionality of the feature space, followed by multiple hidden layers. Each hidden layer incorporates batch normalisation to stabilise training and accelerate convergence. Activation functions are employed to introduce non-linearity, crucial for capturing complex patterns within the data. Selecting Rectified Linear Unit (ReLU) as the activation function in our neural network architecture is justified by its several advantageous properties that contribute to improved training efficiency and model performance. To combat overfitting, which is common in deep neural networks, we employ regularisation techniques such as L1/L2 norms. These penalties are applied to the weights of the network to discourage excessive complexity and improve generalisation capability. Additionally, dropout is strategically inserted between hidden layers to prevent co-adaptation of neurons, thereby enhancing the model's ability to generalise to unseen data.

A critical aspect of our approach involves the meticulous tuning of hyperparameters through an ablation study. We systematically vary parameters such as the number of hidden layers, dropout rates, strengths of L1 or L2 regularisation and learning rates while keeping others constant. This allows us to empirically evaluate their impact on model performance metrics, such as classification accuracy or loss, across different validation sets – the outcomes of the ablation study are presented in Appendix A.

#### k-NN

k-NN is a versatile and intuitive ML algorithm used for both classification and regression tasks.^32^ The core idea behind k-NN is based on the assumption that similar data points are more likely to have the same outcome or belong to the same class. The algorithm works by identifying the ‘k’ nearest neighbours to a given query point from the training dataset, typically using a distance metric such as Euclidean distance. Once the neighbours are identified, k-NN classifies the query point based on the most common class among the neighbours (for classification tasks) or computes the average value (for regression tasks).

One of the primary strengths of k-NN is its simplicity and interpretability, making it particularly useful for problems where the relationship between features and outcomes is complex or non-linear. Unlike many other ML models, k-NN does not require an explicit training phase or a parametric form for the data. This makes it highly flexible, as it can adapt to different data structures without making strong assumptions about the underlying distribution. Additionally, k-NN can easily be visualised, offering transparency into the model's decision-making process. However, k-NN also comes with several limitations that need to be considered. The most prominent issue is its computational cost, especially during the prediction phase. Since the algorithm computes distances between the query point and all training samples, the time complexity increases significantly with larger datasets, making it less efficient for big data applications. Moreover, the choice of the ‘k’ parameter and the distance metric are crucial for the model's performance. A small value of ‘k’ can make the model sensitive to noise, while a large value can smooth over important local variations in the data. Additionally, k-NN is prone to being influenced by irrelevant or redundant features in the data, and it may struggle with high-dimensional or sparse datasets. It is also sensitive to class imbalance, as the majority class may dominate the classification outcome if the neighbours are not evenly distributed.

k-NN relies on the distance between a given test point and all other points in the dataset. To compute this, various distance metrics can be used, such as Manhattan distance, Euclidian distance or in some cases, cosine similarity.^33^ The algorithm then finds the k-nearest points to the training point, with k being user-defined. To classify the point, the algorithm uses majority voting to identify the most commonly assigned class among its neighbours.^34^

In our experiments, we use the scikit-learn implementation of the k-NN classification algorithm.^26^ To choose k, cross-validation was performed based on F1 score and AUCROC, and 18 was chosen as the value of k, which gave a macro (i.e. unweighted) F1 score of 0.79 and an AUCROC of 0.72. We use the Euclidian distance to compute the distance between neighbours.

### Feature importance and risk factors

#### Shapley values and approximations

To identify the risk factors of FA, we employ Shapley values to estimate feature importance in the dataset (namely the importance ranking of the variables that contributes to the risks). Shapley values^12^ are derived from cooperative game theory to offer a theoretically sound and fair method for interpreting the contributions of individual features in a ML model. In the context of ML,^35^ Shapley values are used to attribute the output of a model to its input features, providing a way to interpret the contributions of each feature to the final prediction. More specifically, the Shapley value for a feature represents its average marginal contribution across all possible combinations of features. This ensures a fair distribution of the prediction value among the features based on their contributions, adhering to properties like efficiency, symmetry, dummy and additivity. Importantly, Shapley values provide a model-agnostic method for explaining predictions. This means that they can be applied equally to the five ML models involved in the study to allow a fair comparison. This flexibility is crucial in ensuring that the identified risk factors are consistent and reliable across different types of predictive models. In contrast, feature importance in RF is specific to the RF model, which makes it less flexible compared to Shapley values.

In this research, we experimented with two approaches to approximate the computation of Shapley values. Computing exact Shapley values involves considering all possible subsets of features, which can be computationally expensive, especially for models with a large number of features. Approximation methods such as Kernel SHAP still involve significant amount of computations when the feature set is large – for a dataset with *n* features, this would require *n!* (factorial) evaluations. This quickly becomes infeasible as the number of features increases. Our approaches include zero-input-based Shapley value calculation and permutation-based Shapley value calculation. Both approaches approximate Shapley value computation by focusing on the differences in model output with or without the target features. Consequently, these methods provide a practical balance between computational efficiency and capturing the essential feature importance, albeit with some trade-offs in accuracy. These result in computationally efficient methods for a good approximation of the exact Shapley values. In addition, like Kernel-based methods, both of these methods are model-agnostic, however, unlike Kernel-based methods, it does not require combinatorial masking of all the input variables, hence saving a significant amount of computation. The efficiency of the proposed method makes it more scalable to larger datasets and models. It can handle high-dimensional data and large sample sizes without the prohibitive computational cost associated with permutations.

#### Zero-input vs permutations

The zero-input-based method for evaluating feature importance involves directly setting the target feature value to 0 and then comparing the model's output with and without the target feature. This process starts by calculating the model's prediction using the original dataset with all features included. Next, we create a modified version of the dataset where the target feature is set to 0 for all instances. We then compute the model's prediction using this modified dataset and calculate the difference between the baseline prediction and the modified prediction. This difference is considered the importance of the target feature, with larger differences indicating higher importance.

The permutation-based Shapley value calculation simply approximates Shapley values by calculating values for all permutations of features one-by-one. A variable's contribution is assessed by replacing its value with a randomly drawn value while all other variables keep their real values. The change (if any) in the model's prediction is then noted. A Shapley value is the average expected marginal contribution (in other words, the difference in the model's prediction when the feature is included vs excluded) of one variable after we have tried all combinations.

Theoretically, both the zero-input and permutation-based calculations approximate the exact Shapley value computation by simplifying the process and ignoring the weighting factors on each term. To understand this, we can delve into the function's Taylor series expansion and examine the resulting additive terms. When we expand a function into its Taylor series, we express it as an infinite sum of terms calculated from the values of the function's derivatives at a single point. Each term in the Taylor series represents a contribution to the overall function value, allowing us to see how changes in input variables affect the output. In the context of Shapley value computation, each term in the Taylor series can be grouped into the difference calculated when including or excluding a specific target feature. Both the zero-input-based and permutation-based methods capture this difference by observing the model's output changes when a feature is either set to zero or permuted with other features. This difference highlights the feature's contribution to the model's prediction, aligning with the core idea of Shapley values, which measure the average marginal contribution of a feature across all possible coalitions. However, the exact Shapley value computation involves specific weighting factors for each term, reflecting the probability of a feature appearing in various coalitions. These weights ensure a fair distribution of feature importance by accounting for all possible combinations and their respective probabilities. In contrast, the zero-input and permutation-based methods approximate the Shapley values without incorporating these precise weights. By ignoring the weighting factors, the approximations simplify the computation and reduce the complexity associated with exact Shapley value calculation. While this leads to efficient and scalable methods, it also introduces an approximation error by not fully capturing the nuanced distribution of feature contributions.

We eventually selected to use the zero-input-based Shapley value calculation to identify the feature importance and risk factors in this research. This is due to its interpretability and reduced variance in handling the imbalanced dataset. Directly setting the input feature to zero offers a clear and intuitive way to assess feature importance by representing the feature's absence. This method simplifies the interpretation of results, making it easier for stakeholders to understand the role of each feature in the model's predictions. When we set a feature's value to zero, we simulate the absence of that feature, which can be intuitively understood as removing the influence of that particular variable from the model. This direct manipulation allows us to observe the change in the model's predictions and quantify how much the absence of the feature affects the outcome. For example, if setting a feature to zero significantly alters the model's prediction, it indicates that the feature plays a crucial role in the decision-making process. This method aligns with the real-world scenario of evaluating what happens when a particular factor is not present, making it easier for non-technical stakeholders to grasp the feature's importance. On the other hand, the permutation-based method involves reshuffling the values of a feature while keeping the rest of the dataset intact. This process disrupts the natural correlation between the feature and the target variable but does not completely remove the feature from the dataset. Instead, it creates a scenario where the feature's values are randomised, making it appear as if the feature is still there but without its original context and relationship to other variables. This can be harder to interpret because the feature's influence is not entirely removed but rather obscured by random noise.

Also, the permutation method introduces variance in the calculation of feature importance, and the degree of this variance can be significantly affected by the balance of the dataset. In cases where the data are highly imbalanced – such as when one class has a substantial amount of data while another class has relatively little – the variance introduced by the permutation method can become particularly pronounced. When the dataset is imbalanced, the permutations of features may disproportionately reflect the characteristics of the dominant class. This can lead to an overestimation or underestimation of feature importance for the minority class. Since the permutation method involves reshuffling the values of features and observing the impact on the model's predictions, it inherently captures the underlying distribution of the data. If the majority class dominates, the permutations will mostly reflect the importance of features as they pertain to this class, potentially skewing the results. Moreover, the variance introduced by the permutation method is also a function of the number of permutations performed. In imbalanced datasets, even with a large number of permutations, the likelihood that permutations accurately represent the minority class's feature importance diminishes. This is because the permutations are more likely to be influenced by the majority class's feature distributions. In contrast, the zero-input-based method, by using a fixed alteration (setting to 0), provides a consistent and reproducible measure of feature importance. The zero-input-based method can offer a more consistent measure of feature importance across both classes. These methods reduce the impact of data imbalance on variance since they do not depend on the reshuffling of data points and can provide a more stable estimation of feature importance.

#### Risk factor identification

Our method for evaluating feature importance by setting input features to zero is applied strategically to different patient cohorts to derive targeted insights. We aim to understand the factors influencing both the increase and decrease in A&E attendance among various groups. Here's how we approach this:

- Firstly, we focus on patients who are currently at low and mid-level attendance to A&E. The goal here is to identify the factors that could potentially increase their risk of becoming higher attenders. By setting each feature to zero and observing the changes in the model's predictions, we can pinpoint which features, when absent, lead to significant changes in predicted A&E attendance. These features are identified as key risk factors. Understanding these factors allows us to develop targeted interventions and preventive strategies aimed at minimising the likelihood of these patients increasing their A&E visits.
- On the other hand, we apply the same method to patients who are already at high levels of A&E attendance. Here, the objective is to identify which key risk factors we could target through interventions to reduce their A&E visits. By setting the high-attenders’ risk-related features to zero, we observe which changes lead to a decrease in predicted A&E attendance. These findings highlight the features that, when mitigated, could effectively lower the number of visits. For instance, if reducing the frequency of emergency prescriptions significantly decreases predicted A&E attendance, an intervention might involve better managing chronic conditions through regular outpatient care, thus reducing emergency situations.

This dual application of our method is particularly advantageous when applied to different patient cohorts: those with low and mid-level A&E attendance and those with high attendance, and it provides a comprehensive understanding of the factors influencing A&E attendance across different patient cohorts. For low and mid-level attenders, it identifies potential risk factors for escalation, enabling preventative actions. For high attenders, it highlights actionable risk factors where targeted interventions could lead to a reduction in A&E visits. By tailoring our approach to the specific needs and risk profiles of different patient groups, we can more effectively and accurately identify the factors driving A&E attendance for different patient cohorts. In section ‘Results’, we report the findings on feature importance by aggregating the results from all three categories in Table 2. More detailed results on each individual class is given in Appendix B.

**Table 2.** Combined confusion matrix across the four models.

	Models	Predicted low attendance	Predicted mid attendance	Predicted high attendance	Total
Actual Low attendance	LR	3078	738	245	4061
	RF	3628	400	33	
	SVM	3160	776	125	
	MLP	3871	167	23	
	k-NN	**3937**	**123**	1	
Actual Mid attendance	LR	467	369	243	1079
	RF	829	239	11	
	SVM	**471**	**467**	**141**	
	MLP	815	214	50	
	k-NN	871	198	10	
Actual High attendance	LR	17	14	**61**	92
	RF	40	41	11	
	SVM	21	26	45	
	MLP	**26**	**20**	**46**	
	k-NN	37	37	18	

Bold values denote the highest performing model for each metric.

## Results

### Confusion matrix

A three-class confusion matrix is used to evaluate the performance of a classification model. In this context, the three classes are low, mid attendance and high resource users. The confusion matrix allows us to see how well the model is performing by showing the actual vs predicted classifications. It provides a comprehensive view of the model's performance, highlighting both its strengths and areas for improvement across the three resource usage categories via detailed breakdown for key information including: (a) precision, which measures the proportion of true positive predictions among all positive predictions made by the model. It indicates the accuracy of the positive classifications. High precision means that the model produces few false positives; (b) recall, also known as sensitivity or true positive rate, measures the proportion of true positive predictions among all actual positives in the dataset. It reflects the model's ability to identify all relevant instances. High recall means that the model successfully captures most of the positive cases; and (c) F1 score, which is the harmonic mean of precision and recall, providing a single metric that balances both. It is particularly useful when dealing with imbalanced datasets, as it considers both false positives and false negatives. A high F1 score indicates a good balance between precision and recall. These are the incorrect predictions where the predicted class does not match the actual class, including the number of low resource users incorrectly classified as mid resource users and high resource users, the number of mid resource users incorrectly classified as low resource users and high resource users; the number of high resource users incorrectly classified as low resource users and mid resource users. Together, these numbers indicate the correct classifications and misclassification for each class.

Table 2 combines all the confusion matrices into one table, where the rows represent the actual classes, while the columns represent the predicted classes. For each actual class, the predicted counts for each class are listed for each model. Each count is associated with a model, ensuring clarity in comparison.

Table 3 presents the comparisons of the performance of the four models in precision, recall, F1 and AUC.

**Table 3.** Precision, recall, F1 and AUC of the four models.

	Precision	Recall	F1	AUC
LR	74.06%	67.05%	0.70	**0.77**
RF	74.12%	70.22%	0.72	0.68
SVM	75.01%	70.18%	0.72	0.73
MLP	**75.45%**	**78.96%**	**0.75**	0.73
k-NN	75.57%	79.38%	**0.75**	0.73

Bold values denote the highest performing model for each metric.

The performance of the models is also demonstrated using ROC (Receiver Operating Characteristic) curves. The ROC curve is a graphical plot that illustrates the diagnostic ability of a binary classifier system as its discrimination threshold is varied. It is created by plotting the true positive rate (TPR) against the false positive rate (FPR) at various threshold settings. It shows the trade-off between TPR and FPR across different thresholds. A point in the upper left corner of the plot indicates a good performance. AUC represents the area under the ROC curve. It provides a single scalar value to summarise the overall performance of the classifier. A higher AUC value indicates better overall performance of the classifier. It essentially measures the likelihood that the classifier will rank a randomly chosen positive instance higher than a randomly chosen negative instance.

**Figure 3.**
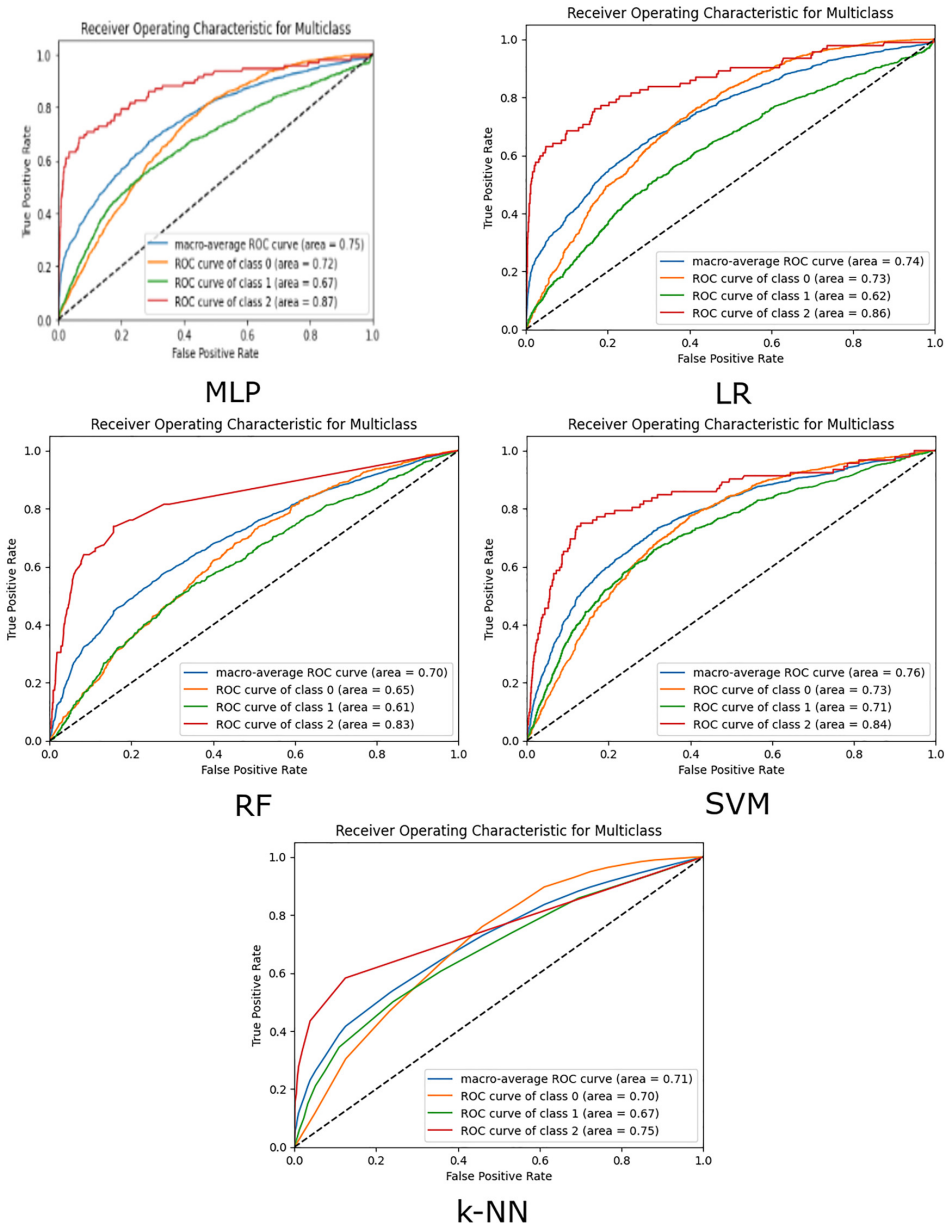
The ROC curves of the five models.

In the three-class classification problem, ROC curves and AUC are adapted to handle multiple classes (Figure 3). The process involves creating and analysing ROC curves for each class separately, using a one-vs-rest approach, and then summarising the performance using the average AUC. For each class, the ROC curve is plotted by considering the current class as the positive class and combining the other two classes as the negative class. This results in three ROC curves, one for each class. Correspondingly, for the three-class problem, we calculate the AUC for each class separately and often average them to get an overall performance metric.

### Discussions

Tables 1 and 2 and Figure 3 illustrate the following main results:

- The k-NN model showed the highest accuracy in predicting low-attendance patients with 3937 correct predictions and the lowest number of misclassifications (123 mid, 1 high). This indicates k-NN's strong capability in identifying low-risk patients accurately. This was followed closely by the MLP model, which correctly predicted 3871 patients as low risk, with incorrect classifications of 167 mid risk and 23 high risk. The RF model also performed well, but with slightly more mid-risk misclassifications. Both LR and SVM had higher misclassifications, indicating their relative weakness in distinguishing low-risk patients.
- In the mid-attendance category, the LR model correctly predicted 369 cases but misclassified 467 cases as low-risk and 243 cases as high-risk. The RF model, on the other hand, correctly predicted 239 cases, while misclassifying a significant number, 829 cases, as low-risk and 11 as high-risk. The SVM model showed best performance in this category with 467 correct predictions, but still had 471 cases misclassified as low-risk and 141 as high-risk. The k-NN model's performance in mid risk is lowest of the five models, correctly classifying 198 cases, while misclassifying 871 cases as low risk and 10 cases as high risk. The MLP model, despite its overall strength, correctly predicted only 214 cases, with 815 misclassified as low-risk and 50 as high-risk. These results highlight the challenge of accurately identifying mid-risk patients, with each model showing varying degrees of misclassification, particularly with a tendency to classify mid-risk patients as low-risk. This suggests that further refinement and potentially more nuanced feature engineering might be necessary to improve mid-risk classification accuracy.
- In the high-risk category, the LR model demonstrated the highest number of correct predictions, accurately identifying 61 high-risk cases while misclassifying 17 cases as low-risk and 14 as mid-risk. The RF model correctly predicted 11 high-risk cases but had a substantial number of misclassifications, with 40 cases identified as low-risk and 41 as mid-risk. Similarly, the k-NN model correctly classifies 18 patients as high-risk, but misclassifies 37 as low-risk and 37 as mid-risk. The SVM model correctly predicted 45 high-risk cases, misclassifying 21 as low-risk and 26 as mid-risk. The MLP model correctly identified 46 high-risk cases, with 26 cases misclassified as low-risk and 20 as mid-risk. These results indicate that while LR was the most successful in correctly identifying high-risk patients, all models struggled with a significant number of misclassifications, particularly between low and mid-risk categories. This underlines the complexity and challenge of accurately predicting high-risk patients, suggesting that further model optimisation and inclusion of additional features may be necessary.

When considering overall precision, recall, F1 score and AUC, MLP stands out with the highest precision (75%), recall (79%) and F1 score (0.75). The MLP model's superior recall indicates its effectiveness in correctly identifying true positive cases across all risk categories. Its higher F1 score reflects a good balance between precision and recall, making it the most reliable model among those tested. The MLP model's superior performance in both correct predictions and lower misclassifications across most of the categories indicates its robustness in capturing the nuanced patterns in our dataset. The architecture of MLP, which allows it to learn complex, non-linear relationships, seems well-suited for our classification task. One reason behind MLP's strong performance is its flexibility to include multiple layers, which enhances its ability to capture intricate feature interactions. Additionally, the use of advanced loss functions such as focal loss and value-hot encoding has likely contributed to its effectiveness in handling imbalanced data. These techniques ensure that the model pays more attention to hard-to-classify examples and high-risk categories, thereby improving overall classification accuracy.

k-NN slightly outperforms MLP with similar precision (76%) and recall (79%) scores, and with an identical F1 score (0.75), and AUC (0.73). However, as shown in the confusion matrix (Table 1), k-NN's performance is dominated by high performance in the low-risk category, while performing the worst in the mid-risk category, and second worst in the high-risk category. Since k-NN relies on majority voting to classify points, it is likely particularly susceptible to the high degree of class imbalance contained in the dataset.

RF showed a strong balance between precision (74%) and recall (70%), with a competitive F1 score (0.72). However, its lower AUC (0.68) suggests some limitations in distinguishing between classes compared to other models. These show that the RF model also performs well, particularly with its low high-risk misclassifications. This performance can be attributed to RF's ensemble nature, which reduces overfitting and improves generalisation by combining multiple decision trees. RF's ability to handle imbalanced data through techniques like bootstrapping and feature bagging also plays a crucial role. These methods help the model to be more robust and less sensitive to noise in the data, allowing it to perform well across different risk categories.

SVM performed well with a precision of 75% and a recall of 70%, resulting in an F1 score of 0.72. Its AUC (0.73) indicates good model performance. LR, while showing reasonable performance, lagged behind the other models with a lower recall (67%) and F1 score (0.70). Its higher AUC (0.77) compared to RF suggests better distinguishing capabilities but still indicates a need for improvement in balancing precision and recall. LR, being a linear model, might not capture the complex interactions between features. It tends to be less flexible compared to non-linear models like MLP and RF, leading to higher misclassifications in mid and high-risk categories. SVM, on the other hand, can handle non-linear boundaries but might struggle with high-dimensional and imbalanced datasets. The standard SVM relies heavily on the choice of kernel and regularisation parameters, which may not be as effective in our context without extensive hyperparameter tuning.

In different clinical scenarios, the preference for high precision or high recall (sensitivity) can vary significantly, especially in the context of A&E attendance. For example, in a scenario focused on prevention, such as identifying patients who might develop severe conditions if not monitored closely, we may want to ensure that all at-risk patients are flagged. In this case, high recall is prioritised to minimise the chance of missing any high-risk patients, even if it means that some low-risk patients are mistakenly identified as high-risk. This approach is crucial for conditions where early intervention can significantly impact outcomes, such as detecting early signs of sepsis or cardiac events, where every potential case must be considered to prevent deterioration. Conversely, there are clinical scenarios in A&E where high precision is preferred. For instance, when dealing with limited resources or when the intervention is invasive or costly, we need to ensure that only those who truly need the treatment are identified as high-risk, thereby avoiding unnecessary risks and side effects associated with the treatment. Here, the goal is to minimise false positives, ensuring that resources are allocated effectively and that patients are not subjected to unnecessary procedures.

A flexible model like MLP allows for these necessary adjustments to balance precision and recall according to specific clinical needs. For example, in A&E, if the focus is on managing chronic conditions that lead to frequent visits, the model can be tuned to have higher recall to catch all potential chronic cases early. This might involve adjusting the architecture to give more weight to features indicative of chronic conditions. On the other hand, if the goal is to reduce unnecessary admissions for non-urgent issues, the model can be adjusted to prioritise high precision, ensuring that only those who absolutely need emergency care are flagged. This can involve refining the loss function and hyperparameters to reduce the FPR, which is particularly useful in managing resources during peak times or pandemics. By having the flexibility to adjust the model's parameters, MLP provides a tailored approach to different clinical requirements, ensuring that the model's performance aligns with the specific goals of the A&E department. This adaptability is essential for addressing the diverse and dynamic nature of emergency medical services, where the balance between precision and recall must be constantly monitored and adjusted to meet the evolving needs of patient care.

Table 4 summarises the performance of ML models for predicting FA and high resource use in healthcare settings varies across studies, reflecting differences in datasets, modelling approaches and classification thresholds. Chmiel et al.^14^ used an XGBoost model on the Southampton Emergency Department dataset (2019–2020), achieving an AUCROC of 0.747 during training. However, the testing precision was relatively low at 0.233, indicating limited accuracy in identifying true positives among predicted positive cases. Pereira et al.^13^ explored multiple models, including AdaBoost, decision trees and logistic regression, using the California Office of State-wide Health Planning and Development dataset (2009–2013). In their three-class classification task, performance metrics varied by frequency group. For low-frequency visits (≤1), AdaBoost achieved a high precision of 0.95 but a moderate sensitivity of 0.61 and AUC of 0.75. For medium-frequency visits (2–4), decision trees showed much lower precision (0.12) and sensitivity (0.40), with an AUC of 0.59. High-frequency visits (≥5) were best identified by AdaBoost with an AUC of 0.84 but very low precision (0.07), highlighting a trade-off between identifying true positives and avoiding false positives. Their binary classification task using AdaBoost demonstrated a strong AUC of 0.93 for a threshold of ≥9 visits, yet sensitivity remained a challenge. Grinspan et al.^10^ applied models such as lasso, RF and AdaBoost, achieving high AUCROC values (0.7) but low sensitivity (<50%) across all methods. This indicates that while the models were good at ranking patients based on their likelihood of FA, they struggled to capture all true positive cases.

**Table 4.** Model performance in other research.

Study	Methods	Population	Results
Chmiel et al. 2021^14^	XGBoost	Southampton Emergency Department 2019–2020	AUCROC of 0.747 during training; precision of 0.233 during testing.
Pereira et al. 2016^13^	AdaBoost, decision trees, logistic regression	California Office of State-wide Health Planning and Development dataset (2009–2013)	Three-class classification: low frequency (≤1 visit) AdaBoost sensitivity: 0.61, precision: 0.95, AUC: 0.75. Medium frequency (2–4 visits) decision tree sensitivity: 0.40, precision: 0.12, AUC: 0.59. High frequency (≥5 visits) AdaBoost sensitivity: 0.61, precision: 0.07, AUC: 0.84. Binary classification: AdaBoost (AUC 0.93 with ≥9 visit threshold).
Grinspan et al. 2015^10^	Lasso, random forests, AdaBoost	Health information exchange in New York City, epilepsy patients	High AUCROC (0.7) but low sensitivity (<50%) across all methods.

In comparison, our models demonstrated more balanced and robust performance across all metrics. For example, our MLP model achieved the highest F1 score (0.75) and recall (78.96%), indicating superior ability to balance precision and recall. It also maintained a strong AUC of 0.73, showing reliable ranking capability. Logistic regression (LR) and SVM also performed consistently well, with LR achieving an F1 score of 0.70 and an AUC of 0.77, while SVM demonstrated a slightly better balance between precision (75.01%) and recall (70.18%). The k-NN model, showed competitive performance, with an F1 score of 0.74, precision of 75.20% and recall of 78.72%. Unlike previous studies that often struggled with sensitivity, our models, particularly MLP and k-NN, excelled in recall while maintaining reasonable precision. This suggests that our approach, which incorporates advanced methods like Shapley value approximation for feature importance and carefully tuned hyperparameters, is better suited to the complex and imbalanced nature of A&E frequent attendance data. Our comprehensive comparison across five models further strengthens the generalisability of our findings and provides actionable insights for clinical decision-making.

### Risk factors identified via Shapley values

Table 5 shows consensus and differences of the risk factors identified by the four different models. We use columns to show the feature ranking (in descending order) for all the four models. The importance (i.e. Shapley) values are displayed next to the features, indicating their positive or negative contributions to the risks.

**Table 5.** Comparisons of the risk factors (with an absolute importance value > 0.01) identified by the models.

Importance	LR	RF	SVM	k-NN	MLP
(0.2 0.3)	Acute (0.24)	Acute (0.28)		Acute (0.37)	Acute (0.31)
(0.1 0.2)		Digestive (0.15)	Acute (0.11)		
(0.01 0.1)	Gender (0.08) Digestive (0.07) Respiratory (0.04) Alcohol (0.02) Self-harm (0.02) NeuroLTC (0.01) Mental (0.01) Homeless (0.01)	Alcohol (0.05) Gender (0.04) Respiratory (0.04) Self-harm (0.03) Mental (0.02) Diabetes (0.02)	Digestive (0.05) Gender (0.04) Respiratory (0.03) Self-harm (0.02) Diabetes (0.02) Homeless (0.02) Mental (0.02)	Digestive (0.04) Self-harm (0.03) Alcohol (0.02) Respiratory (0.02) Mental (0.01)	Gender (0.08) Digestive (0.07) Alcohol (0.05) Respiratory (0.03) Mental (0.02) Self-harm (0.02) Diabetes (0.01) Oth LTC (0.01) NeuroLTC (0.01)
(−0.1 −0.01)	Home care (−0.01) Cardiac (−0.01) Cancer (−0.01) SIMD (−0.06)	Cancer (−0.01) Deceased (−0.02) Home care (−0.02) SIMD (−0.08)	Substance (−0.02) Home care (−0.02)	Deceased (−0.02) SIMD (−0.1)	Cancer (−0.01) Cardiac (−0.01) SIMD (−0.04)
(−0.2 −0.1)	Deceased (−0.18) Age (−0.19)	Age (−0.20)	SIMD (−0.15) Deceased (−0.16)		Deceased (−0.14) Age (−0.14)
(−0.3 −0.2)			Age (−0.30)		
(−0.4 −0.3)				Age (−0.31)	

Table 5 shows the following common risk factors identified across the four models:

- Acute inpatient episodes: Identified as a top risk factor by all five models, with values ranging from 0.11 (SVM) to 0.37 (k-NN). The prominence of acute conditions across all models indicates that recent acute medical issues are strongly associated with increased A&E visits.
- Gender: Consistently identified as an important risk factor by all models except k-NN, with importance values ranging from 0.04 (RF, SVM) to 0.08 (MLP). This suggests that gender plays a significant role in predicting frequent A&E attendance (namely more male than female), potentially due to underlying differences in health-seeking behaviour and medical needs.
- Digestive conditions: Highlighted by all models, with values from 0.05 (SVM) to 0.15 (RF). Digestive issues are commonly associated with emergency medical needs, thus frequent A&E visits.
- Respiratory conditions: Present in all models, though the importance values are generally lower, ranging from 0.03 (SVM, MLP) to 0.04 (RF, LR). This consistency underscores the role of chronic respiratory conditions in driving emergency healthcare utilisation.
- Self-harm: Identified by all models, with importance values from 0.02 (SVM, LR and MLP) to 0.03 (RF, k-NN). This reflects the critical need for emergency services in cases involving self-harm.
- Mental health issues: Another common factor, though the importance varies, with values from 0.01 (LR, k-NN) to 0.02 (RF, SVM and MLP). The frequent need for emergency intervention for mental health crises is likely the reason for this trend.

In addition, other risks factors that are positively contribute to the high resources risk, as identified by most of the models are:

- Alcohol: Highlighted by LR (0.02), k-NN (0.02), RF (0.05) and MLP (0.05), but not by SVM. This discrepancy might be due to SVM's sensitivity to high-dimensional data and its specific feature selection process, which may not prioritise alcohol consumption as highly as the other models.
- Homelessness: Identified by LR (0.01) and SVM (0.02), but not by RF, k-NN or MLP. The complex social and medical needs of homeless individuals could be captured differently by each model, particularly since RF and MLP might better integrate other contributing factors.
- Diabetes: Recognised by RF (0.02), SVM (0.02) and MLP (0.01) but not by LR or k-NN. LR's linear nature may not capture the multifaceted impacts of diabetes as effectively as the more flexible models.

The rationale behind these outcomes is as follows: Positive contributors to frequent A&E visits include factors such as admissions due to alcohol misuse, self-harm and mental health inpatient episodes. High alcohol consumption often leads to acute medical crises, such as injuries, overdoses and liver disease complications, necessitating emergency care. Individuals engaging in self-harm are at immediate risk of severe injury and require urgent medical attention, making self-harm a significant predictor of frequent A&E visits. Similarly, mental health issues often result in crises that require immediate intervention to ensure patient safety, contributing to higher A&E attendance. Some specific health conditions such as digestive and respiratory conditions contribute to the increase of A&E visits. Also, the acute nature of medical conditions and their potential for sudden and severe health impacts make them key indicators of the need for emergency medical services.

Conversely, certain conditions and factors contribute negatively to the risk of frequent A&E visits, including:

- SIMD: Consistently identified as a significant negative factor across all models, with values ranging from −0.04 (MLP) to −0.15 (SVM). This suggests that higher poverty levels (lower SIMD values) are associated with higher A&E visits.
- Deceased: Identified by LR (−0.18), RF (−0.02), k-NN (0.02), SVM (−0.16) and MLP (−0.14). This negative importance is intuitive, as deceased individuals no longer contribute to A&E visits.
- Age: Consistently a significant negative factor, with values from −0.14 (MLP) to −0.31 (k-NN). This suggests that older age groups may have fewer A&E visits, potentially due to better management of chronic conditions or reduced likelihood of acute events leading to emergency visits.
- Home care: Identified negatively by LR (−0.01), RF and SVM (−0.02). This could indicate that individuals receiving home care have reduced need for emergency visits due to regular monitoring and management of their conditions.
- Cancer: Identified negatively by LR (−0.01), RF (−0.01) and MLP (−0.01), but not significantly by SVM. This negative importance might reflect the stable management of cancer patients, reducing their emergency visits.
- Cardiac conditions: Negative importance identified by LR (−0.01) and MLP (−0.01), but not by RF or SVM. This could indicate effective outpatient management reducing emergency visits for these conditions.

The rationale behind these outcomes is as follows: despite being a severe condition, cancer is managed through structured and continuous care outside of emergency services, reducing the necessity for frequent A&E visits. Many cancer patients have planned admissions for treatment rather than unplanned emergency visits. Similarly, chronic cardiac conditions are typically managed through regular outpatient visits and planned interventions, which reduce the frequency of emergency visits. Improved medical management and regular monitoring can help maintain stability, thus lowering emergency needs for cardiac patients.

The SIMD, which represents the multifactorial socio-economic gradient, consistently emerges as a significant negative factor across all models. This suggests that higher poverty levels result in higher A&E visits. This highlights the complex interplay between socio-economic factors and healthcare utilisation.

Age is another significant negative factor, with older adults potentially having fewer emergency visits due to stable management of chronic conditions, greater use of planned healthcare services or different health-seeking behaviours. Regular monitoring and management of health conditions through home care services also contribute negatively to the risk of frequent A&E visits. These services provide continuous care, reducing the need for emergency interventions and leading to fewer A&E visits.

Understanding these positive and negative contributors helps in tailoring interventions and resource allocation to manage frequent A&E attenders effectively. The identification of these risk factors provides valuable insights into which populations are at higher or lower risk of frequent A&E visits, guiding targeted strategies for prevention and management.

### Comparisons with other research in Scotland

This study is broadly comparable with similar studies in the literature. It covers most of the variables used by other research, ensuring a comprehensive analysis that incorporates a wide range of relevant factors. This inclusivity allows for a more robust comparison and understanding of the various influences on frequent A&E attendance. Meanwhile, our study presents several unique aspects that distinguish it from previous research in the field. In particular, we present our focus on identifying high resource users (i.e. >10 A&E visits per year). Other unique characteristics include: (a) the study is conducted at a specific location, namely the A&E at NHS Lanarkshire to provide detailed insights into the local population and healthcare dynamics to inform targeted interventions and policies within this region; (b) the dataset used in this study is unique in that it only includes attendance from three and above, excluding those with fewer visits. This focus on higher-frequency attenders allows for a more concentrated analysis of the factors contributing to repeated A&E usage; and (c) finally, this study involves models that have been used in other studies, such as LR, RF and SVM, which shows consistent performance with the findings of this research. In addition, we have placed more emphasis on the MLP model. By focusing on MLP, the study aims to explore the effects of state-of-the-art deep learning techniques on predicting frequent A&E attendance, providing insights into their potential advantages and limitations compared to more traditional models. Our study reveals that the performance of deep neural networks can vary significantly due to their flexible architecture and the variety of loss functions available. Our research demonstrates that with careful design of the model architecture and strategic selection of hyperparameters, DNNs can achieve superior performance compared to the other three models. This underscores the importance of leveraging the versatility of deep neural networks to optimise predictive accuracy in risk identification.

Our study identifies several risk factors similar to the findings from previous research carried out also in Scotland, such as the importance of mental health, socio-economic factors, gender and some chronic conditions. However, our findings also diverge in some areas, particularly concerning the role of age and cancer conditions, highlighting the unique aspects of our dataset and the need for further investigation to reconcile these differences. (1) Kyle et al.^16^ focused on groups predominantly composed of males, identifying mental health and substance misuse problems as common risk factors. Their findings align with our study, where mental health were also highlighted as significant risk factors. Additionally, both studies found a strong association between FA and socio-economic deprivation. Our study, however, does not identify substance misuse as a key risk factor, but includes a broader set of other variables such as respiratory and digestive conditions, which were not specifically mentioned in Kyle et al.'s work. (2). Wyke et al.^17^ associated high attendance in a General Practice setting with a greater number of serious conditions and higher levels of anxiety but did not find a direct link to socio-economic conditions. In contrast, our study identified socio-economic deprivation (measured through SIMD) as a significant negative risk factor. Moreover, while anxiety was not explicitly measured in our study, mental health issues were a common risk factor, which could implicitly cover aspects of anxiety. (3). Cruwys et al.^18^ found FA to be associated with chronic conditions such as overweight and obesity, high blood pressure and drug prescriptions, along with the potential influence of social group connections. Our study similarly highlighted chronic conditions like respiratory issues as important risk factors. However, our data did not explicitly investigate social group connections, which Cruwys et al. suggested might be a critical area for further research. (4). Finally, the previous study in Lanarkshire NHS found that 90% had at least one LTC with 37% having more than five conditions, and 77% of frequent attenders lived in the most deprived areas (SIMD 1 and 2). The outcomes of this research have largely confirmed these previous findings.

Some possible explanations of the discrepancy is as follows:

- Age group was found to be negatively correlated with higher A&E attendances in this dataset. While this conflicts with some of the findings of existing literature, potential explanations present themselves. In this dataset, mental health, substance misuse and alcohol misuse are all significant factors in explaining higher frequent attendance. These factors tend to be associated with younger age groups.^^32,36^^ In addition, the vast majority of A&E admissions in this dataset were for under 65s. This would seem to suggest that the high number of admissions for the above mentioned conditions in NHS Lanarkshire causes younger people to make up a higher percentage of admissions, and therefore results in older age not being a significant factor in predicting higher attendance.
- Additionally, gender was found to be highly correlated with higher A&E attendances in this dataset, despite the majority of A&E attendances in Lanarkshire being for patients of male gender. Our literature review found that the value of gender as a predictor of FA was inconsistent in existing literature. More research is likely needed to explore the relationship between gender and FA, and this is left to future work.
- Chronic conditions such as cancer and cardiovascular disease have been identified as negative contributors. This negative importance might reflect the stable management of these conditions.

## Conclusions

In conclusion, this research has used ML models for the prediction of FA in NHS Lanarkshire patients. Here we conclude this research as follows:

Firstly, we examine which model has best performance on the dataset. The MLP model's outstanding performance across most of the risk categories underscores its flexibility and adaptability in handling complex and imbalanced data. This can be attributed to its ability to leverage deep learning techniques and advanced loss functions, which are crucial for capturing the intricate patterns in the dataset. k-NN also performed strongly on the dataset across most metrics, but this performance was dominated mainly by high performance in the low-risk category. This results in k-NN presenting itself as a model which captures the trade-off between precision and recall well, but one which may not be robust to high class imbalance. k-NN's highly interpretable nature means that it remains a strong contender with unique benefits. The SVM models also demonstrated commendable performance, particularly in the mid-risk category where it exhibited a balanced precision and recall. SVM's robustness in high-dimensional spaces and its capacity to handle non-linear boundaries with kernel tricks make it a valuable model. Despite having slightly higher misclassifications in the low-risk category compared to MLP, SVM maintained competitive overall performance, indicating its effectiveness in capturing complex patterns in the data. The RF model also shows strong performance, particularly in handling high-risk predictions, due to its ensemble approach and robustness to overfitting. Logistic regression, while performing reasonably well, highlights the limitations of linear and traditional ML approaches in modelling the complex dependencies present in the data. However, its strength in identifying high-risk cases and its simplicity and interpretability still hold value, particularly when complemented with more advanced techniques like MLP. Overall, the combination of MLP's advanced architecture and tuning capabilities, RF's robust ensemble approach, SVM's balanced performance, k-NN's high performance and simplicity and LR's interpretability provides a comprehensive understanding of the risk prediction landscape. Each model offers unique strengths that can be leveraged to improve predictive accuracy and reliability in different contexts.

Also, our study demonstrated strong and balanced performance across multiple metrics (precision, recall, F1 and AUC) when compared to existing research on frequent attenders. Unlike previous studies, which often reported imbalanced outcomes, our models provide consistent scores across all metrics. These findings suggest that our approach offers a more comprehensive and adaptable framework for predicting FA and identifying risk factors, addressing gaps in prior research and setting a benchmark for future studies.

This analysis also demonstrates that certain health conditions and risk factors consistently predict frequent A&E attendance across different models such as the acute inpatient episodes, gender, digestive, respiratory conditions, self-harm and mental health issues and alcohol, while other factors vary in their importance. The differences in model selection and the complexity of interactions between features highlight the need for diverse approaches in predicting healthcare utilisation. Understanding these patterns helps in tailoring interventions and resource allocation to manage frequent A&E attenders effectively. The identification of both positive and negative risk factors provides insights into which populations are at higher or lower risk of frequent A&E visits, guiding targeted strategies for prevention and management.

Lastly, we examine the similarity of our results with results of previous research. The results of this research are largely comparable with existing studies, although there are some differences that highlight unique aspects of our dataset and methodology. Similar to Kyle et al.^16^ and Cruwys et al.,^18^ our research identifies mental health issues as significant risk factors for FA in A&E. Both studies, along with ours, underscore the influence of socio-economic deprivation on attendance rates. However, while Wyke et al.^17^ did not find socio-economic conditions to be directly associated with high attendance, our study identified socio-economic deprivation as a significant risk factor. Additionally, while Mills et al. (2022)^19^ highlighted age and cancer type as key factors among those diagnosed with cancer, our research found age and cancer to negatively contribute to the risk, suggesting that older age and cancer diagnoses are associated with lower attendance. These differences may stem from variations in study populations, timeframes and specific variables considered, but overall, the core findings on the importance of mental health, chronic conditions and socio-economic factors are consistent across studies.

### Limitations of the study and future work

The current study has several limitations that must be acknowledged to understand the context and potential constraints of our findings. Firstly, the dataset used covers only the period from 2021 to 2022, which coincides with the COVID-19 pandemic. The pandemic significantly altered healthcare utilisation patterns, with many people avoiding hospitals due to infection fears or lockdown measures. Consequently, the frequent attendance patterns observed during this period might not be representative of more typical times. The generalisability of our results to non-pandemic periods remains uncertain, and future studies should incorporate data from both pre- and post-pandemic periods to validate these findings.

Additionally, our dataset exclusively includes patients with three or more A&E visits, omitting those with one or two visits. This focus on FA means that we lack information on the majority of A&E users, who typically have fewer visits. Understanding the characteristics and risk factors of this larger group could provide a more comprehensive picture of A&E utilisation patterns and might reveal different predictors of attendance frequency. Including this broader spectrum of attendance data in future research would enhance the completeness and applicability of the findings.

Another significant limitation is the lack of detailed external information, such as comprehensive social status indicators beyond the SIMD. While SIMD is a valuable measure of socio-economic status, it does not capture all the nuances of an individual's social circumstances. Other factors, such as employment status, educational background and detailed housing conditions, can provide deeper insights into the social determinants of health and their impact on A&E attendance. Incorporating these additional variables in future studies would likely yield a more holistic understanding of the factors influencing FA.

One area of future work which presents itself is the possibility of utilising AI/ML approaches to develop consistent risk scores for patients, and the ability to generate these risk scores in real time. This would allow AI/ML to be integrated into the clinical processes involved in assessing FA as a decision support tool. One avenue of investigation for the development of real-time assessments of risk scores is the use of time-series data.

Further to this, the evaluation of prevention and early intervention approaches requires more investigation. For a risk score to be truly effective, health services must be aware of which options for treatment are likely to be effective in lowering the risk score.

In conclusion, while this study provides valuable insights into the risk factors associated with frequent A&E attendance, it is limited by its temporal scope, the exclusion of those attending less frequently and the lack of comprehensive social data. Future research should address these limitations by incorporating a broader range of data, both temporally and demographically, and by including more detailed socio-economic indicators. Additionally, potential AI/ML approaches should focus on providing actionable information such as real-time risk scores which are consistent across patients, and prevention and early intervention strategies should be fully explored. This will help to validate and extend our findings, making them more generalisable and actionable for healthcare providers and policymakers.

## Appendix

### Appendix A. An ablation study of the MLP architecture and hyperparameters

An ablation study was conducted to evaluate the performance of a neural network model by systematically altering its architectural components and hyperparameters. The baseline model configuration (default) included two layers with batch normalisation, a weighted focal loss function adjusted according to the number of data samples, L1 regularisation, a dropout rate of 0.5 and a learning rate of 0.001. The performance of this baseline model was assessed using precision, recall, F1 score, AUC and accuracy.

To understand the impact of different architectural and hyperparameter choices, various modifications were made to the baseline configuration. Specifically, the study explored the following variations:

- Layer depth: The number of layers was increased from 2 to 11.
- Loss function: The focal loss was replaced with a weighted loss function without the focal component.
- Regularisation: L1 regularisation was substituted with L2 regularisation.
- Dropout rate: The dropout rate was varied to 0, 0.2 and 0.7.
- Learning rate: The learning rate was adjusted to 0.0001 and 0.01.

Increasing the number of layers to 11 provided insights into the benefits and drawbacks of deeper networks. While deeper layers can capture more complex patterns, they might also lead to overfitting if not properly regularised. Using a weighted loss without the focal component tested the importance of focusing on hard-to-classify. This change helped determine the effectiveness of the focal loss in handling class imbalances. Substituting L1 with L2 regularisation evaluated the impact on model generalisation. L2 regularisation can help mitigate overfitting by penalising larger weights differently than L1. Varying the dropout rate allowed us to study its effect on preventing overfitting. A higher dropout rate generally increases robustness, while too much dropout can hinder learning. Adjusting the learning rate tested the model's sensitivity to this critical hyperparameter. A smaller learning rate often results in more stable convergence, while a larger rate can speed up learning but may cause instability.

The results of these experiments, detailed in Table A.1, reveal how each change impacts the neural network's performance metrics. The baseline model performance include:

- Precision: The baseline model achieved a high precision, indicating its accuracy in predicting positive cases.
- Recall: The model showed strong recall, demonstrating its ability to identify most positive instances.
- F1 score: A high F1 score reflected a good balance between precision and recall.
- AUC: The model's AUC indicated excellent overall performance in distinguishing between classes.
- Accuracy: The baseline model maintained high accuracy, reflecting its overall reliability.

The ablation study, as summarised in Table A.1, highlights the trade-offs involved in neural network design and provides a comprehensive understanding of how different configurations influence performance. This study underscores the importance of careful model tuning to achieve optimal results in precision, recall, F1 score, AUC and accuracy.

**Table A.1.** The outcomes of an ablation study of the MLP architecture and hyperparameters (I).

Hyperparameter	Configurations	Precision	Recall	F1 score	AUC	Accuracy
Baseline	Default	74.76%	71.39%	0.73	0.76	71.39%
Layers	3	73.81%	71.16%	0.72	0.77	71.16%
	4	72.59%	70.09%	0.70	0.73	70.09%
	5	73.07%	73.34%	0.72	0.74	73.34%
	6	73.41%	72.92%	0.72	0.75	72.92%
	7	72.36%	72.65%	0.70	0.74	72.65%
	8	75.27%	77.62%	0.73	0.74	77.62%
	9	73.66%	76.09%	0.71	0.75	76.09%
	10	74.39%	77.12%	0.71	0.74	77.12%
	11	74.70%	76.93%	0.71	0.72	76.93%
Loss	Weighted	73.31%	75.38%	0.71	0.76	75.38%
Regularisation	L2	71.50%	74.25%	0.71	0.73	74.25%
Dropout rate	0	73.52%	64.97%	0.68	0.73	64.97%
	0.2	73.51%	70.89%	0.72	0.72	70.89%
	0.7	74.40%	78.44%	0.70	0.73	78.44%
Learning rate	0.0001	73.17%	78.02%	0.69	0.67	78.02%
	0.01	73.53%	78.50%	0.71	0.61	78.50%

A second ablation study was conducted to further investigate the performance of the neural network, with a primary focus on the impact of the loss function configuration. In this study, the baseline model utilised value-hot cross entropy with focal loss, which adjusts the loss contribution based on the attendance number, adding a higher penalty for misclassifying high attendance cases. Similar to the first study, the baseline model featured two layers with batch normalisation, L1 regularisation, a dropout rate of 0.5 and a learning rate of 0.001. The main variation in this second study involved evaluating the model's performance without incorporating the focal loss component. This adjustment allowed us to assess the significance of the focal loss in handling class imbalances and its overall effect on precision, recall, F1 score, AUC and accuracy. Other aspects of the ablation study, such as variations in layer depth, regularisation, dropout rates and learning rates, mirrored those in the first study and are not repeated here. The findings, summarised in Table A.2, highlight the importance of focal loss in enhancing the model's ability to manage imbalanced data and improve predictive performance across multiple metrics.

**Table A.2.** The outcomes of an ablation study of the MLP architecture and hyperparameters (II)

Hyperparameter	Configurations	Precision	Recall	F1 score	AUC	Accuracy
Baseline	Default	75.13%	71.98%	0.73	0.76	75.13%
Layers	3	74.88%	72.99%	0.74	0.76	72.99%
	4	73.89%	75.73%	0.74	0.73	75.73%
	5	73.54%	75.54%	0.73	0.72	75.54%
	6	**75.45%**	**78.96%**	**0.75**	**0.73**	**78.96%**
	7	74.42%	78.46%	0.73	0.73	78.46%
	8	75.40%	79.01%	0.74	0.70	79.01%
	9	71.30%	78.31%	0.70	0.71	78.31%
	10	71.43%	78.19%	0.70	0.74	78.19%
	11	71.87%	78.13%	0.69	0.72	78.13%
Loss	Value hot	75.02%	77.92%	0.75	0.77	77.92%
Regularisation	L2	75.04%	78.36%	0.75	0.72	78.36%
Dropout rate	0	73.41%	64.20%	0.68	0.74	64.20%
	0.2	74.21%	71.56%	0.72	0.75	71.56%
	0.7	75.72%	78.96%	0.73	0.69	78.96%
Learning rate	0.0001	74.99%	79.03%	0.73	0.68	79.03%
	0.01	73.26%	78.48%	0.73	0.54	78.48%

Bold denotes values of model which was chosen.

### Appendix B. Results of risk factor identification

Tables B.1 to B.3 present the results of applying Shapley value approximation to determine the importance of various risk factors for three distinct cohorts: low attendance, mid attendance and high resource users. This analysis provides insights into the different factors that contribute to the risk of frequent attendance and high resource use, helping us understand the unique characteristics of each group.

**Table B.1.** Comparisons of the risk factors (with an absolute importance value > 0.01) for people with low attendance, as identified by the models.

Importance	LR	RF	SVM	k-NN	MLP
(0.2 0.3)		Acute (0.27)	Acute (0.25)	Age (0.25)	
(0.1 0.2)	Acute (0.18) Digestive (0.1)	Digestive (0.19)		SIMD (0.14)	Acute (0.18) Gender (0.14)
(0.01 0.1)	Gender (0.05) Respiratory (0.02)	Gender (0.06) Mental (0.02) Alcohol (0.01)	Digestive (0.07) Alcohol (0.03) Respiratory (0.02) Gender (0.02) Mental (0.01) Oth LTC (0.01)	Deceased (0.03) Home care (0.02)	Digestive (0.09) Respiratory (0.02) Oth LTC (0.01) Alcohol (0.01) NeuroLTC (0.01)
(−0.1 −0.01)	Cardiac (−0.01) Home care (−0.01) SIMD (−0.09)	Deceased (−0.01) Home care (−0.02)	Home care (−0.02) SIMD (−0.09)	Respiratory (−0.01) Diabetes (−0.01) Alcohol (−0.02) Gender (−0.03)	Cancer (−0.02) Cardiac (−0.01) SIMD (−0.05)
(−0.2 −0.1)		SIMD (−0.13)	Deceased (−0.13)	Digestive (−0.1)	Age (−0.16)
(−0.3 −0.2)	Deceased (−0.21) Age (−0.24)	Age (−0.24)	Age (−0.29)	Acute (−0.35)	Deceased (−0.2)

**Table B.2.** Comparisons of the risk factors (with an absolute importance value > 0.01) for people with medium attendance, as identified by the models.

Importance	LR	RF	SVM	k-NN	MLP
(0.5 0.6)				Age (0.6)	
(0.4 0.5)					
(0.3 0.4)					
(0.2 0.3)					
(0.1 0.2)	Acute (0.19) Digestive (0.1)	Acute (0.14) Digestive (0.11)			Acute (0.19)
(0.01 0.1)	Gender (0.08) Digestive (0.07) Respiratory (0.05) NeuroLTC (0.02) Alcohol (0.02) Mental (0.01)	Respiratory (0.08) Alcohol (0.06) Gender (0.05) Mental (0.03) Diabetes (0.02) Self-harm (0.02) NeuroLTC (0.02)	Gender (0.04) Digestive (0.04) Respiratory (0.04) Diabetes (0.03) Alcohol (0.02) Mental (0.02) Self-harm (0.02) Homeless (0.01) Oth LTC (0.01)	SIMD (0.1) Home care (0.05) Gender (0.02)	Gender (0.08) Digestive (0.08) Respiratory (0.04) Alcohol (0.03) Oth LTC (0.02) Mental (0.02) Diabetes (0.02) NeuroLTC (0.01)
(−0.1 −0.01)	Arthritis (−0.01) Home care (−0.01) Cardiac (−0.01) Cancer (−0.02) SIMD (−0.05)	Deceased (−0.01) Home care (−0.03) Cancer (−0.03) SIMD (−0.04)	Home care (−0.01) Acute (−0.02)	Alcohol (−0.01) Self-harm (−0.01) Mental (−0.02) Diabetes (−0.02) Respiratory (−0.05) Acute (−0.07)	Home care (−0.01) Cardiac (−0.01) Cancer (−0.02) SIMD (−0.04)
(−0.2 −0.1)	Deceased (−0.19)		SIMD (−0.16) Deceased (−0.18)		Deceased (−0.15) Age (−0.15)
(−0.3 −0.2)	Age (−0.21)				
(−0.4 −0.3)		Age (−0.33)	Age (−0.36)		

**Table B.3.** Comparisons of the risk factors (with an absolute importance value > 0.01) for people with high resource use, as identified by the models.

Importance	LR	RF	SVM	k-NN	MLP
(0.5 0.6)				Acute (0.58)	
(0.4 0.5)					
(0.3, 0.4)	Acute (0.35)	Acute (0.35)			Acute (0.39)
(0.1 0.2)		Digestive (0.14)			Alcohol (0.1)
(0.01 0.1)	Gender (0.08) Digestive (0.07) Digestive (0.05) Self-harm (0.05) Alcohol (0.05) Respiratory (0.03) NeuroLTC (0.02) Homeless (0.01)	Alcohol (0.08) Self-harm (0.06) Respiratory (0.05) Gender (0.03) Diabetes (0.03) Oth LTC (0.02) Mental (0.02)	Gender (0.09) Acute (0.06) Self-harm (0.06) Respiratory (0.04) Digestive (0.03) Diabetes (0.02) NeuroLTC (0.02) CereLTC (0.01) Mental (0.02)	Self-harm (0.07) Alcohol (0.04) Substance (0.02) NeuroLTC (0.02) Mental (0.01) CereLTC (0.01)	Self-harm (0.05) Digestive (0.04) Mental (0.04) Gender (0.03) Respiratory (0.02) Substance (0.02) Diabetes (0.01)
(−0.1 −0.01)	SIMD (−0.03)	Cardiac (−0.01) Home care (−0.02) Deceased (−0.02) SIMD (−0.06) Age (−0.09)	Home care (−0.01) Substance (−0.05) Alcohol (−0.08)	Digestive (−0.01) Deceased (−0.02) Home care (−0.02) Diabetes (−0.03) SIMD (−0.03)	SIMD (−0.03) Deceased (−0.09) Age (−0.13)
(−0.2 −0.1)	Deceased (−0.12) Age (−0.13)		Deceased (−0.14) Age (−0.16) SIMD (−0.18)	Age (−0.11)	

Table B.1 shows the Shapley value rankings for low attendance individuals. The risk factors in this category include acute conditions, gender and digestive issues, among others. This table highlights the importance of these factors in predicting low attendance patterns, with acute conditions having the highest Shapley value, indicating its significant contribution to this cohort.

Table B.2 displays the Shapley value rankings for mid attendance individuals. In this category, factors such as acute conditions, gender and digestive issues remain important, but other factors like mental health and self-harm also emerge as significant contributors. This table demonstrates how the importance of risk factors shifts as attendance frequency increases, reflecting the changing dynamics in mid attendance patterns.

Table B.3 presents the Shapley value rankings for high resource users. Here, acute conditions, gender and digestive issues continue to be important, but additional factors like alcohol use and chronic conditions gain prominence. This table underscores the complexity of predicting high resource use, with a broader range of risk factors playing a critical role in this cohort.

Overall, these tables illustrate the varying impact of different risk factors across the three attendance categories. By using Shapley value approximation, we can effectively rank the importance of these factors, providing valuable insights for designing targeted interventions and prevention strategies tailored to each cohort's specific needs.

### Appendix C. Rapid literature review summary

**Table C.1.** Summary of rapid literature review.

Study	Objectives	Location	Area	Methods
Acosta and Lima (2015)^37^	Identify profile, factors, reasons for frequent use of emergency services	Brazil	National	Mixed-methods: descriptive statistics, Fisher's exact test, Spearman's correlation, semi-structured interviews, thematic content analysis
Chen et al. (2020)^38^	Examine sociodemographic and visit characteristics of high system users	Canada	National	Numerical summaries (means, SDs, IQR), logistic regression models, multivariable logistic regression
Beck et al. (2016)^39^	Identify characteristics of ED attendees with mental health referrals	UK	London	Cox proportional hazards regression, negative binomial regression
Cordell et al. (2022)^40^	Identify proportion and characteristics of frequent ED presenters	Australia	Sydney	Descriptive statistics, Pearson's chi-square test, Fisher's exact test, Mann–Whitney test, multivariate logistic regression
Burns (2017)^41^	Review of literature on factors for frequent ED use	USA	St Louis	Narrative synthesis
Cho et al. (2023)^42^	Identify characteristics and factors for frequent ED use	South Korea	National	Frequency analysis, chi-square test, multiple logistic regression
Daniels et al. (2018)^43^	Characteristics and unmet needs of frequent ED attenders	UK	Bath	Mixed-methods: qualitative interviews, descriptive statistics, business data, case note analysis
Castillo et al. (2014)^44^	Compare hospital-specific vs community-wide identification of frequent users	USA	San Diego	Retrospective cohort study, comparisons between identification methods, confidence intervals
Dufour et al. (2020)^45^	Factors associated with frequent ED use among older adults	Canada	Quebec	Descriptive statistics, chi-square test, Kruskal–Wallis test, multivariate regression model
Colligan et al. (2016)^46^	Examine factors for persistent frequent ED use in Medicare beneficiaries	USA	National	Multinomial logistic regression
Chiu et al. (2020)^9^	Estimate prevalence and factors of persistent frequent ED use; compare characteristics with occasional frequent users	Canada	National	Multivariable logistic regression; odds ratios
Hotham et al. (2021)^8^	Test hypothesis: main variables for ED attendance are multimorbidity and increasing age	UK	National	Logistic regression; entropy and count of presenting complaint categories
Diaz et al. (2014)^7^	Compare likelihood of frequent GP attendance between natives and immigrants; study associated socio-economic and morbidity factors	Norway	National	Multivariate binary logistic analyses; binary logistic regression
Burton et al. (2021)^47^	Test hypothesis: frequent attendance can be understood as a complex system	UK	National	Analysis of health records; fitting data to power law distributions
Shukla et al. (2020)^48^	Suggest uniform definition of frequent attenders; determine correlated factors	USA	National	Systematic literature review
Byrne et al. (2003)^49^	Describe frequent attenders’ general health service use, clinical, psychological and social profiles	Ireland	National	Matched case-control study; questionnaires
Paul et al. (2010)^6^	Determine factors associated with frequent ED attendance in Singapore	Singapore	National	Stepwise logistic regression analysis
Jelinek et al. (2008)^50^	Examine characteristics of adult patient attendances to EDs by frequency of attendance	Australia	Perth	SPSS analysis, χ^2^ test, Student's *t*-test
Afonso and Lopes (2020)^51^	Identify ED high-frequency users and compare their clinical and utilisation characteristics with other ED users	Portugal	National	Kruskal–Wallis test, Spearman correlation, chi-squared tests
Chang et al. (2014)^52^	Identify patient characteristics associated with frequent ED use	USA	Greater Boston	Descriptive statistics, chi-square tests, multivariate logistic regression
Palmer et al. (2014)^53^	Determine if having regular access to a primary care provider affects frequency of ED use	Canada	Saint John	Descriptive statistics, χ^2^ tests, logistic regression
Chan et al. (2018)^54^	Describe characteristics of ED visits by FAs to a hospital in Singapore	Singapore	National	Logistic regression
Castillo et al. (2019)^55^	Evaluate patient characteristics and patterns of ED use among geriatric patients	USA	California	Descriptive statistics, logistic regression, odds ratios
Moore et al. (2009)^56^	Identify personal and attendance factors associated with ED attendance frequency	UK	National	Frequencies, cross-tabulations
Al-Saffar et al. (2020)^57^	Identify sociodemographic and clinical characteristics of children who attend primary care frequently	UK, Croatia, Spain, USA	Multinational	Systematic review, narrative synthesis
Blair et al. (2017)^58^	Describe sociodemographic and clinical characteristics of pre-schoolers who attend ED frequently	UK	National	Descriptive analyses, Poisson regression
Pek et al. (2022)^59^	Quantify extent of multiple ED use by FAs and characterise FAs	Singapore	National	Chi-square tests, Mann–Whitney U test, multivariable logistic regression
Chou et al. (2021)^60^	Determine impact of COVID-19 on healthcare-seeking behaviours among frequent ED users	Taiwan	National	Descriptive statistics, Mann–Whitney U test
Jacob et al. (2016)^20^	Characterise FAs, especially those with medically unexplained and mental health symptoms	UK	National	Descriptive statistics, case note review
Sousa et al. (2019)^61^	Evaluate impact of FA service on number and frequency of ED attendances	UK	Durham	Descriptive statistics, independent sample *t*-test
Aagaard et al. (2014)^62^	Identify predictors of frequent use of a psychiatric emergency room	Denmark	National	Logistic regression, semi-structured interviews
